# Frontocingulate-parietal-limbic circuits associated with both ruminative brooding and self-regulatory processes

**DOI:** 10.3389/fnhum.2026.1731382

**Published:** 2026-03-13

**Authors:** Selena Singh, Vibooshitha Thusyanthan, Allison Mizzi, Yarden Levy, Isaac Kinley, Saurabh Bhaskar Shaw, Suzanna Becker

**Affiliations:** 1 Department of Psychology, Neuroscience and Behaviour, McMaster University, Hamilton, ON, Canada; 2 Nova Scotia Health Authority, Halifax, NSW, Canada; 3 Kaplan and Levitt Psychologists, Hamilton, ON, Canada; 4 Rotman Research Institute, Baycrest Academy for Research and Education, Toronto, ON, Canada; 5 Data Sciences Institute, University of Toronto, Toronto, ON, Canada; 6 Department of Psychiatry, Western University, London, ON, Canada

**Keywords:** brooding, cross-frequency coupling, emotional regulation, metacognition, mindfulness, neural networks, symptom networks

## Abstract

**Introduction:**

Ruminative brooding is a transdiagnostic symptom defined as repetitive dwelling on thoughts and emotions, and is linked to emotion dysregulation, maladaptive metacognitive beliefs, and abnormal interoception. The relative contributions of these factors and their neural mechanisms remain unclear. In this exploratory study, we mapped these processes onto directed cross-frequency coupling (CFC) networks using resting-state electroencephalography.

**Methods:**

We first identified symptoms of interest for CFC analyses by employing regularized symptom networks, revealing two clusters relevant to brooding: one dominated by interoceptive/mindfulness dimensions and another by metacognitive/emotional dysregulation, with brooding belonging to the latter. We then examined links between representative symptoms from each cluster and resting-state cross-frequency phase–amplitude coupling (PAC) using partial least squares correlation (PLS-C).

**Results:**

Emotional dysregulation and brooding dimensions co-varied with delta-beta PAC (representing a “brooding/dysregulation” neural signature), whereas mindfulness symptoms co-varied with beta-gamma and theta-gamma PAC (representing a “mindfulness/interoception” neural signature). More specifically, for the brooding/dysregulation signature, prefrontal and cingulate phase activity modulated amplitudes in regions implicated in emotion regulation and interoception. In contrast, the mindfulness/interoception signature reflected coupling within circuits supporting emotion regulation/interoception.

**Discussion:**

Overall, our results indicated that brooding was more tightly linked to maladaptive metacognitive beliefs and emotional dysregulation than to mindfulness/interoception, consistent with resistance toward one’s thoughts and emotions. Neurally, as reflected through multivariate PLS-C covariance patterns, this may be linked to compensatory top-down control from prefrontal and cingulate areas over interoceptive, affective, and self-referential systems, pointing to the potential value of therapies that cultivate self-acceptance and modify maladaptive metacognitive beliefs for reducing rumination.

## 1 Introduction

Rumination is a transdiagnostic symptom common to mood, anxiety, trauma-related and sleep disorders, to name a few (Ehring and Watkins, 2008; Watkins, 2009). This symptom involves repetitive dwelling on thoughts, moods or past events (Nolen-Hoeksema, 2000). In depression, ruminative thought content is often negative and self-deprecating, separating into two distinct subtypes: ruminative brooding and reflective pondering (Treynor et al., 2003). These two subtypes differ in content and functional significance. Reflective pondering is analytical in nature, involves problem-solving to understand causes of negative mood, and is thought to be *adaptive* as it *negatively* predicts future depressive episodes (Treynor et al., 2003). Ruminative brooding, on the other hand, involves the passive dwelling on abstract and critical thoughts, and is considered the *maladaptive* form, as it predicts future depressive episodes, depression severity and recurrence (Joormann et al., 2006; Treynor et al., 2003). The cognitive and underlying neural mechanisms of ruminative brooding remain unclear; understanding these mechanisms could help inform treatment selection and design.

Mechanistic accounts of rumination, and other forms of repetitive negative thinking such as worry in anxiety, implicate both “bottom-up” emotion-driven processes and/or “top-down” cognitive control mechanisms (Ikani, 2021). “Bottom-up” processes are typically automatic in nature, requiring little cognitive effort as they are driven by cognitive biases and emotions. In contrast, “top-down” processes require effort and rely on cognitive systems such as those involved with attention, working memory, and cognitive control. While previous work has mainly focused on rumination from a “top-down” perspective, revealing deficits in inhibitory control (Singh et al., 2025; Whitmer and Banich, 2007), poor working memory updating (Joormann and Gotlib, 2008; Zetsche et al., 2012), and attentional disengagement (Koster et al., 2011; Whitmer and Gotlib, 2013), the role of “bottom-up” factors remains unclear.

Integrative constructs that combine *both* top-down and bottom-up processes, such as emotional regulation, mindfulness, interoceptive awareness, and metacognitive beliefs, have been independently linked to rumination. Each of these constructs reflects how an individual relates and responds to their inner experiences, and have significant top-down (e.g., appraisal, cognitive control) and bottom-up (e.g., affect- and sensory-driven) components. These factors play a significant role in rumination, especially given that initial conceptualizations of rumination in both anxiety and mood disorders highlight this process as a stress-reactive emotional regulation process, in which individuals repetitively evaluate the causes and meanings of their moods and thoughts (Davey, 2006; Mennin et al., 2005; Nolen-Hoeksema, 1991, 2000). It is therefore crucial to consider mechanisms that integrate both top-down and bottom-up processes when studying rumination.

Mindfulness involves deliberately focusing one’s attention on current internal and external experiences, observing thoughts and emotions without judgment (Chems-Maarif et al., 2025; Kabat-Zinn, 1994). Relatedly, interoceptive awareness reflects an individual’s awareness of their internal bodily sensations (e.g., heartbeat, organ function, respiration, satiety) along with autonomic nervous system activity related to emotions (Barrett et al., 2004; Cameron, 2001; Craig, 2002; Vaitl, 1996). Low mindfulness and poor interoceptive awareness have been linked to heightened ruminative brooding (Alleva et al., 2014; Lackner and Fresco, 2016), reflecting difficulties with emotion regulation and, in turn, heightened depression and anxiety symptoms (Lackner and Fresco, 2016). Notably, some individuals have reported heightened sensitivity to interoceptive cues (e.g., awareness of their heartbeat) during episodes of rumination (Schlinkert et al., 2020). These mixed results may be explained by stress response system (SRS) dysregulation theories, such as the Allostatic Load Model, in which initial stress exposure leads to hypervigilance, increasing awareness of interoceptive cues (Del Giudice et al., 2011; Ellis et al., 2011; Juster and Misiak, 2023). Chronic stress exposure, however, may excessively burden the SRS and lead to subsequent down-regulation (Del Giudice et al., 2011; Juster and Misiak, 2023), leading to insensitivity to internal states and their causes (Schultchen et al., 2019). Rumination after acute vs. chronic stress exposure may therefore be differentially related to interoceptive cue sensitivity. Regardless of an individual’s trait interoceptive sensitivity, mindfulness- and interoceptive awareness-based treatments aim to balance one’s awareness of their internal states while encouraging reappraisal and goal-directed behaviour change, ultimately reducing distress (Price and Hooven, 2018). Mindfulness-based therapies have been shown to reduce rumination by improving attentional control and promoting *adaptive* awareness of bodily sensations (Hammerdahl et al., 2025; Heeren and Philippot, 2011; Perestelo-Perez et al., 2017; van der Velden et al., 2023).

The role of metacognitive beliefs in rumination both complements and diverges from mindfulness and interoceptive awareness. Like mindfulness and interoception, metacognitive processes involve heightened awareness of internal experiences, yet they diverge in being explicitly evaluative: whereas mindfulness emphasizes nonjudgmental observation and interoceptive awareness emphasizes sensitivity to bodily states, metacognition entails the appraisal, monitoring, and control of one’s thoughts (Flavell, 1979; Moritz and Lysaker, 2018). Metacognitive beliefs represent relatively stable assumptions about the meaning and function of one’s cognitive processes. These beliefs can either be adaptive (e.g., “catastrophizing is unhelpful”), or maladaptive (e.g., “worrying now will help me later”), with the latter being consistently linked to psychopathology (Capobianco and Nordahl, 2023). According to one account, repetitive negative thinking, including depressive rumination, is reinforced by maladaptive metacognitive beliefs concerning the function and consequences of such thinking (Wells and Matthews, 1996) (e.g., “If I didn’t ruminate about my feelings of depression, they would take over me/never end” (Papageorgiou and Wells, 2001)). Subsequent work supported this account, showing that individuals with recurrent depression often endorsed rumination as a coping strategy to manage overwhelming emotions, while also perceiving it as uncontrollable and thus contributing to feelings of personal failure (Papageorgiou and Wells, 2001). Importantly, interventions designed to modify maladaptive metacognitive beliefs have been shown to reduce rumination across multiple psychopathologies (Sharma et al., 2022), highlighting the importance of maladaptive metacognitive beliefs in rumination.

Although these constructs have been studied in isolation in brooding, their relationship to brooding after considering construct *interdependence* has not yet been investigated. Mindfulness and metacognitive beliefs may be functionally related, with evidence demonstrating that high mindfulness is associated with less dysfunctional metacognition, likely due to reducing the negative appraisal of internal thoughts (Solem et al., 2017). In addition, metacognition may facilitate mindfulness by enabling the monitoring of internal experiences (Jankowski and Holas, 2014; Norman, 2017). However, it remains unclear which of these factors plays the most salient role in rumination. A promising approach for studying the interdependence between these factors, along with identifying central features for ruminative brooding, is by estimating regularized partial correlation networks (also known as “symptom” or “psychological” networks) (Epskamp et al., 2018; Epskamp and Fried, 2018). These networks are composed of nodes (the “symptoms”) and edges (strength of mutual associations between symptoms); the resulting structure reveals relationships and patterns between symptoms while controlling for all others. Although these networks are primarily used to map relationships between symptoms of a disease or condition (i.e., at the population level), this approach also offers a valuable exploratory approach for identifying candidate features of interest for subsequent analyses. Specifically, here we are interested in linking these candidate features of rumination to their neural substrates; the potential to ground these psychological processes in brain networks and circuits not only provides us with information on the neural underpinnings but may also reveal possible targets for neurostimulation treatments (e.g., repetitive transcranial magnetic stimulation, or rTMS, and deep-brain stimulation).

Electroencephalography (EEG) offers a non-invasive method for identifying neural substrates of cognitive phenomena, while also enabling analyses that require high temporal resolution, such as those involving neural oscillations. Neural oscillations span a wide range of frequency bands, with each thought to play a distinct functional role (Fries, 2015). Furthermore, the functional *coupling* between oscillations of different frequency bands, known as cross-frequency coupling (CFC), is thought to be a marker of large-scale coordination between and within brain networks (Canolty and Knight, 2010). CFC occurs when the phase or amplitude of one oscillation is functionally coupled to the phase or amplitude of another. For example, phase-amplitude CFC occurs when the amplitude of a high-frequency oscillation is synchronized and/or modulated by the phase (e.g., the peak or trough) of a low-frequency oscillation. CFC may facilitate cross-brain region coordination by enabling two oscillations, localized to different brain areas, to interact. When computed across pairs of brain areas, CFC can be used to construct functional connectivity networks.

CFC features have been linked to emotional regulation, mindfulness/interoceptive awareness, and cognitive functions. Interestingly, distinct CFC signatures are thought to reflect bottom-up and top-down processes (Fries, 2015). Delta (2–4 Hz)-beta (13–30 Hz) coupling has been presented as a marker of emotional (dys)regulation (Myruski et al., 2022; Poppelaars et al., 2021) and a neural predictor of cortisol response under stress (Wang X. et al., 2024). Theta (4–8 Hz) oscillations have been implicated across many functions, including mindfulness (Duda et al., 2024; Lomas et al., 2015), with theta-gamma (30–80 Hz) CFC involved in functions relevant to ruminative brooding, including memory, attention, and emotion (Ursino and Pirazzini, 2024). Both trait mindfulness/interoceptive awareness and rumination have been associated with beta oscillations in brain regions involved with self-referential processing and attentional control (Benschop et al., 2021; Ferdek et al., 2016; Ng et al., 2021). The role of beta activity in supporting these seemingly opposing processes (i.e., mindfulness and rumination) remains unclear. One possibility is that the brain accommodates both by reconfiguring functional networks according to current demands (Reinhart and Woodman, 2014), for example, by switching the directionality of oscillatory coupling. Since gamma oscillations are thought to reflect local circuit computations (Fernandez-Ruiz et al., 2023; Fries, 2009), studying beta-gamma CFC will enable the investigation of information flow within rumination- and mindfulness-related circuits. Given this putative function of gamma oscillations, the beta phase may reflect modulatory influences, whereas the gamma amplitude may capture localized computations, enabling identification of regions that act as “modulators” vs. “processors” within these networks. Using CFC to compute functional connectivity networks therefore enables the study of how slower rhythms in one region organize fast local computations in another, capturing neurocomputationally relevant directed information *flow* that single-band connectivity measures typically obscure. Examining functional networks derived from CFC may therefore offer a novel method for linking symptom network organization with ruminative neural dynamics.

There are a number of regions distributed amongst the default mode, salience, central executive, and sensorimotor networks that may be relevant for brooding, emotion regulation, interoception, metacognition and mindfulness (Table 1). Notably, both the posterior parietal cortex and posterior cingulate cortex have been heavily implicated in ruminative brooding (Andersen et al., 2009; Benschop et al., 2021; Bocharov et al., 2021a; Fink et al., 1996; Forner-Phillips et al., 2020a; Kircher et al., 2002), with these regions forming a circuit with medial temporal lobe structures, such as the parahippocampal gyrus, supporting autobiographical memory recall and self-referential processes (Aminoff et al., 2013; Kobayashi and Amaral, 2003; Leech and Sharp, 2014; Suzuki and Amaral, 1994). Mindfulness and interoceptive awareness may require brain regions integrating interoceptive and somatosensory cues, such as the insula (Berntson and Khalsa, 2021; Cauda et al., 2011; Khalsa et al., 2009) and somatosensory cortex (Cameron and Minoshima, 2002; Critchley et al., 2004; Tamè et al., 2016). Both metacognition and mindfulness, along with the processing of bottom-up emotional cues, may require the top-down appraisal of thoughts facilitated by the dorsolateral prefrontal cortex, which is well known to be involved in executive functions (Friedman and Robbins, 2022). Finally, many of these regions, such as prefrontal and limbic areas, work together to facilitate emotional regulation and processing (Banks et al., 2007; Delgado et al., 2008; Johnstone et al., 2007; Siegle et al., 2007; Urry et al., 2006). For example, the subcallosal cingulate plays an important role in negative emotional processing (Hamani et al., 2011; Vogt, 2014; Vogt and Vogt, 2009), and has been associated with dorsolateral prefrontal cortex activity in depression (Benschop et al., 2022). How these sub-circuits interact to facilitate both brooding and self-regulatory processes remains unclear. We propose that investigating this question through CFC networks may be a useful first step.

**Table 1:** ROI Destrieux labels and justification for inclusion.

Region	Abbreviation	Destrieux label(s)	Functional relevance
Anterior cingulate cortex	ACC	*G_and_S_cingul-Ant*	Part of the limbic system and the salience network. Known to play a role in both cognitive control and emotional stability (Bush et al., 2000).
Posterior cingulate cortex	PCC	*G_cingul-Post-dorsal* *G_cingul-Post-ventral*	Involved in self-referential processing and rumination (Benschop et al., 2021; Fink et al., 1996; Kircher et al., 2002). Strongly connected to the parahippocampal gyrus and entorhinal cortex, therefore related to the hippocampal memory system (Kobayashi and Amaral, 2003; Leech and Sharp, 2014; Suzuki and Amaral, 1994).
Subcallosal cingulate cortex (also known as subgenual cingulate)	SCC	*G_subcallosal*	Involved in emotion regulation and is heavily implicated in depression (Hamani et al., 2011; Vogt, 2014) and previously associated with rumination in remitted major depression (Benschop et al., 2021).
Parahippocampal gyri	PHG	*G_oc-temp_med-Parahip*	Episodic memory (Aminoff et al., 2013) and depressive symptoms (Zamoscik et al., 2014)
Insula	Insula	*G_Ins_lg_and_S_cent_ins* *G_insular_short*	Emotional processing and interoceptive awareness (Berntson and Khalsa, 2021; Cauda et al., 2011; Khalsa et al., 2009)
Ventromedial prefrontal cortex	vmPFC	*S_orbital_med-olfact* *S_orbital-H_Shaped* *G_orbital*	Emotion regulation of negative emotions (Delgado et al., 2008; Johnstone et al., 2007; Urry et al., 2006), self-referential processing (D’Argembeau, 2013) and autobiographical memory recall (McCormick et al., 2020)
Dorsolateral prefrontal cortex	dlPFC	*G_front_middle* *S_front_middle* *S_front_sup*	Executive functions (Friedman and Robbins, 2022), and top-down regulation of limbic activity (Banks et al., 2007; Siegle et al., 2007). Compensatory connectivity at rest demonstrated in depression (Iseger et al., 2017; Wang Y. et al., 2024).
Somatosensory cortex	SSC	*G_postcentral*	Interoceptive awareness, sensory perception and integration, and bodily awareness (Cameron and Minoshima, 2002; Critchley et al., 2004; Tamè et al., 2016).
Posterior Parietal cortex	PPC	*G_pariet_inf-Supramar* *G_parietal_sup*	Working memory, attention, spatial cognition (Marek and Dosenbach, 2018), and autobiographical memory (Brown et al., 2018). Has been implicated in rumination (Andersen et al., 2009; Bocharov et al., 2021b; Forner-Phillips et al., 2020b).

The present study aims to explore the neural correlates of brooding and concurrent self-regulatory processes to move towards an integrative neural theory. We address this aim through the following two approaches: (1) by presenting a symptom network analysis of mindfulness, interoceptive awareness, metacognitive beliefs and ruminative brooding to identify potential patterns of interdependence among symptoms (e.g., clustering signatures) used primarily to guide subsequent EEG analyses; and (2) by linking those symptom network-derived features with functional connectivity markers from CFC-derived neural networks. We aim to test the hypotheses that (1) ruminative brooding is associated with diminished mindfulness and heightened maladaptive metacognitive beliefs, and that (2) similar circuits will underlie mindfulness, metacognition, and ruminative brooding, differing in the frequency of oscillations participating in CFC and directionality of the frequency pairing. By bridging ruminative brooding-related symptoms with functional neural networks, the present study aims to explore CFC patterns that reflect the interaction between top-down and bottom-up processes. Characterizing these network dynamics and symptom interactions may help to guide future research by identifying targets for neuromodulatory or cognitive interventions that enhance regulatory control and reduce ruminative brooding.

## 2 Methods

### 2.1 Experiment

#### 2.1.1 Study design and participants

The data used for the analyses presented in this paper are from a larger study on the neural dynamics of depressive rumination. This larger study included questionnaire measures of rumination, mood, emotional regulation, mindfulness, interoceptive awareness, dissociation, metacognitive beliefs, sleep (described in detail below), along with handedness; a cognitive control assessment via the Stroop task; and finally 5 min of resting-state EEG followed by a task-switching EEG protocol adapted from a previous study from our lab (Shaw et al., 2021). Participants completed rumination and mindfulness measures in person, prior to the EEG session, and completed the remaining self-report measures using a take-home survey. The present study includes results from the resting state EEG data only, as well as a selection of questionnaire measures from the full study (i.e., all questionnaires except for the handedness assessment). We focussed on the resting state EEG data only for this study, as the task-based EEG data was aimed at testing different hypotheses regarding dynamical systems in rumination, which are fundamentally unrelated to the aims of the present study.

We aimed to recruit 40 undergraduate students through McMaster University’s SONA research participant recruitment system. We decided on this sample size based upon previous studies of rumination including EEG connectivity, source-localization and spectral analyses, which have reported samples ranging between *N =* 26–45 (Bocharov et al., 2021a; Ferdek et al., 2016; Forner-Phillips et al., 2020a; Reiser et al., 2012). Our exclusion criteria included a self-reported history of traumatic brain injury, any current or previous mental health diagnoses (e.g., major depressive disorder, bipolar disorder, post-traumatic stress disorder, etc.), and current engagement in more than 5 min of weekly mindfulness practice. We excluded participants with >5 min of weekly mindfulness practice as we aimed to assess mindfulness as an inherent, trait-like feature, rather than one that has been actively learned or manipulated by the participants over time. All participants provided informed consent, and protocols were approved by the McMaster Research Ethics Board (MREB number 5987). We collected data from 48 participants, of whom all provided complete symptom data, and 31 provided complete and usable EEG data (i.e., correctly saved, loadable EEG files, with data demonstrating repairable artifact contamination and/or noise). The sample comprised individuals aged 18–25, of whom 60% identified as female, 11% as male, and 29% did not report their sex and/or gender.

#### 2.1.2 Questionnaire measures

We assessed trait-level rumination, depression, anxiety and stress levels using self-report measures as follows. Our primary measure of interest, ruminative brooding, was assessed using the brooding subscale of the Ruminative Response Scale (RRS) (Nolen-Hoeksema, 2000; Nolen-Hoeksema and Morrow, 1991). Although we aimed to recruit participants without mental health diagnoses, it is possible that some exhibited subclinical symptoms of depression or anxiety (Beiter et al., 2015), or had not yet received a formal diagnosis. In addition to the RRS, we therefore used the Depression Anxiety Stress Scale (DASS-21) to assess levels of depression and anxiety symptoms along with perceived chronic stress (Lovibond and Lovibond, 2011). The DASS-21 is grounded in a dimensional model of depression, anxiety, and stress, which views differences between clinical and nonclinical populations as variations in symptom severity. The DASS-21 cannot, therefore, provide a clinical *diagnosis* of depression, anxiety or a stress-related disorder, but can be helpful to probe the severity of psychopathological symptoms. We therefore used the recommended cut-off scores to assess symptom severity (i.e., normal, mild, moderate, severe and extremely severe) (Lovibond and Lovibond, 2011).

To measure integrative (i.e., top-down and bottom-up) mechanisms of rumination, including metacognitive beliefs, emotional regulation, interoceptive awareness and mindfulness, we used the following self-report measures. To assess participant metacognitive beliefs, we included the Metacognitions Questionnaire (MCQ) to assess the following beliefs: positive beliefs about worry, negative beliefs about worry, cognitive confidence, need to control thoughts, and cognitive self-consciousness (Cartwright-Hatton and Wells, 1997). Furthermore, we assessed emotional regulation using the Difficulties in Emotional Regulation Scale (DERS), which consists of 6 subscales: nonacceptance of emotional responses, difficulties engaging in goal-directed behaviour, impulse control difficulties, lack of emotional awareness, limited access to emotional regulation strategies, lack of emotional clarity (Gratz and Roemer, 2004).

To measure participant levels of trait interoceptive awareness and mindfulness, we included the following measures: the second version of the Multidimensional Assessment of Interoceptive Awareness (MAIA-2) (Mehling et al., 2018) and the Five Facet Mindfulness Questionnaire (FFMQ-39) (Baer et al., 2006; Shallcross et al., 2020). The MAIA-2 consists of 8 subscales: noticing (awareness of uncomfortable, comfortable and neutral body sensations); non-distracting (tendency not to ignore or distract oneself from sensations of pain or discomfort); not-worrying (tendency not to worry or experience emotional distress with sensations of pain or discomfort); attention regulation (ability to sustain and control attention to body sensations); emotional awareness (awareness of the connection between body sensations and emotional states); self-regulation (ability to regulate distress by attention to body sensations); body listening (active listening to the body for insight); and trusting (experience of one’s body as safe and trustworthy). The FFMQ-39 consists of 5 subscales: observing, describing, acting with awareness, non-judging of inner experience, and nonreactivity to inner experience. Given the similarities between rumination and dissociation in terms of a lack of connection to the present environment, we also assessed trait dissociation using the total score from the second version of the Dissociative Experiences Scale (DES-II) (Carlson and Putnam, 1993).

Along with mindfulness/interoceptive awareness, emotional regulation, depression and anxiety, rumination is also known to interfere with sleep (Morin et al., 2003); as such, sleep quality may be a marker of physiological distress. We assessed participant sleep quality using the Sleep Quality Scale (SQS), comprising 6 subscales: daytime dysfunction, restoration after sleep, difficulty falling asleep, difficulty getting up, satisfaction with sleep, and difficulty maintaining sleep (Yi et al., 2006).

#### 2.1.3 Resting state EEG acquisition

Resting-state EEG data were collected for 5 min using a BIOSEMI ActiveTWO system with 128 wet, gel-based electrodes in a sound-attenuated, dimly lit room. Since active electrodes provide impedance transformation on the electrode, generating an output impedance of <1 *Ω*, the level of DC offset is typically used to evaluate quality of electrode contact rather than impedance values. We ensured that electrode offsets were kept within ± 20 μV. Data were collected at a sampling rate of 2048 Hz. Participants were instructed to close their eyes and minimize movement for the duration of the 5 min.

### 2.2 Subscale analyses

#### 2.2.1 Feature selection for symptom networks

Features derived from symptom network analyses are sensitive to sample size, with large samples (i.e., N > 100) typically required to support adequate stability analyses (Epskamp et al., 2018). Due to our relatively small sample size for this analysis (*N =* 48), we decided *a priori* to select the top 15 symptoms that were the most predictive of participant brooding scores using a regularized regression (“Elastic Net”) (Zou and Hastie, 2005). This initial regression analysis was conducted in Python using the *scikit-learn* package (v. 1.3.2), and was not aimed at analyzing predictors of ruminative brooding, but rather, at identifying symptoms with the highest-magnitude non-zero coefficients for subsequent analyses. Elastic Net regression combines both lasso and ridge regularization to shrink very small (i.e., irrelevant) coefficients by placing a penalty term in front of coefficients during model training as a part of the loss function. *Lasso* shrinks very small coefficients to zero, which is excellent for feature selection, but may erroneously shrink some non-zero coefficients if there is a high degree of multicollinearity between measures (e.g., between our measures of depression, stress, and rumination, or mindfulness and interoceptive awareness). *Ridge* regularization will shrink coefficients evenly without eliminating them, which is a good strategy for handling multicollinearity, but is not ideal for feature selection purposes. Elastic net combines both of these regularization techniques, enabling feature selection while handling multicollinearity (Zou and Hastie, 2005). The Elastic Net regression model was trained using 5-fold cross-validation on the standardized symptoms to predict ruminative brooding. The mixing parameter was set to 0.5 to balance the L1 (lasso) and L2 (ridge) penalties equally.

**Figure 1:**
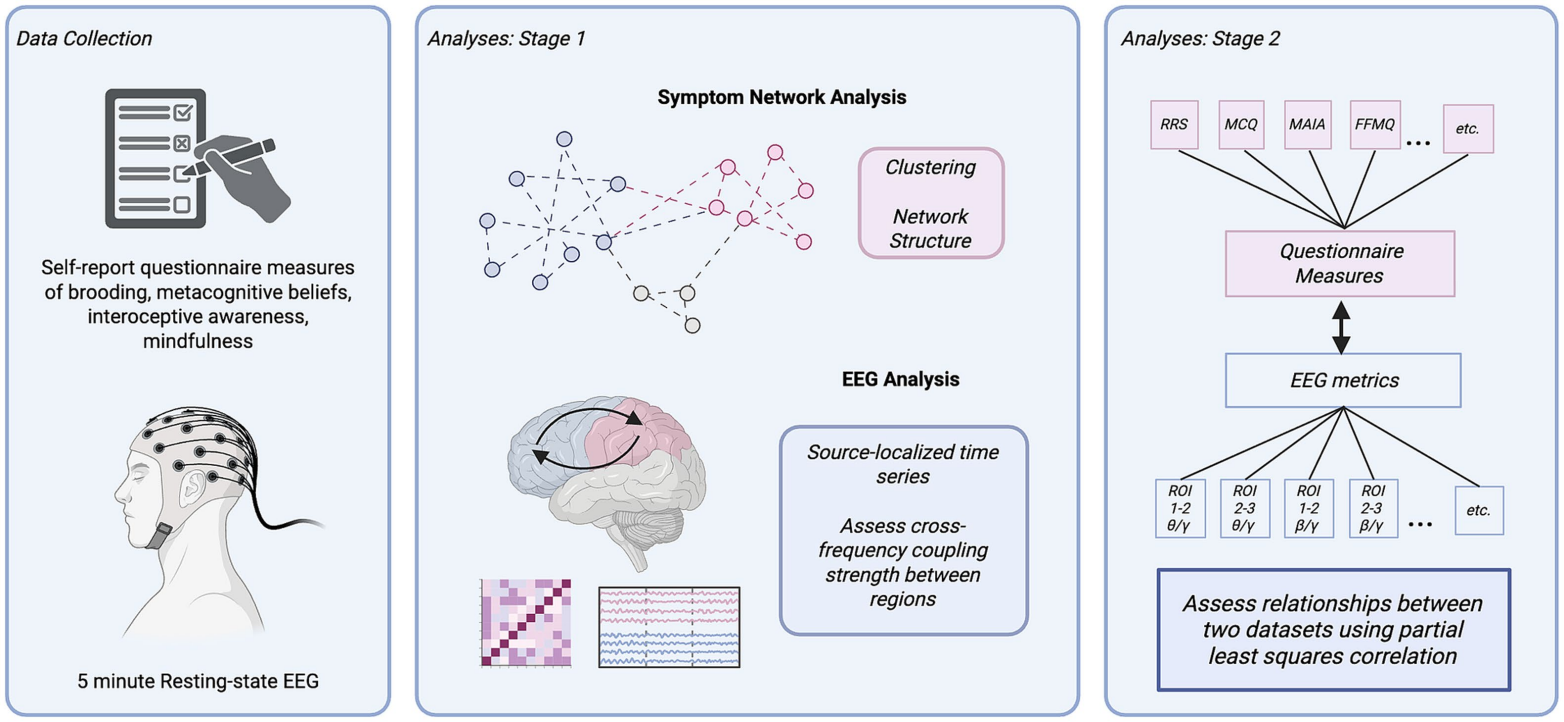
Overview of study methodology. Participants first completed questionnaire measures of rumination, metacognitive beliefs, interoceptive awareness, and mindfulness, followed by 5 min of resting-state EEG using a 128-lead system. Relationships among symptoms were analyzed by constructing regularized partial correlation networks (“symptom networks”) and assessing network structure and clustering behaviour of symptoms. EEG data were analyzed by first performing source localization to map signals onto regions of interest (ROIs), followed by assessing phase–amplitude cross-frequency coupling between theta-gamma (*θ*/*γ*), delta-beta (*δ*/*β*), and beta-gamma (β/γ) oscillatory bands. During the second stage of analysis, a set of symptoms from the symptom network was selected and mapped onto cross-frequency coupling scores using partial least squares correlation.

Our features of interest included the subscale and total scores of the RRS, DASS-21, DERS, MAIA-2, MCQ, FFMQ-39, DES-II, and SQS. Both total and subscale scores were entered as predictors, as Elastic Net is ideally suited to adjudicate between correlated variables to identify whether *overall* severity or *specific* symptom dimensions contributed more strongly to the prediction of brooding, as indexed by their retention and relative coefficient magnitude in the Elastic Net model. We excluded the DASS-21 and RRS depression subscales as the RRS brooding subscale may already be confounded with depression symptomatology (Treynor et al., 2003). In total, we assessed 39 scale-based scores as predictors of brooding severity. We selected symptoms with the top 15 largest magnitude regression coefficients for our subsequent exploratory network analyses (Figure 1).

#### 2.2.2 Estimating symptom networks

We estimated sparse Gaussian graphical models using the extended Bayesian information criterion applied to the graphical lasso (EBICglasso) algorithm (Epskamp and Fried, 2018). We implemented these models using the *bootnet* and *psych* packages in R (Epskamp and Fried, 2018). This algorithm involves first computing a correlation matrix across the 15 previously selected symptoms, followed by applying a Lasso penalty on the inverse covariance matrix to encourage sparsity. 100 network configurations were assessed, with the EBIC applied to choose the best-fitting sparse model (prioritizing goodness of fit and model simplicity while accounting for the small sample size). We used a moderate EBIC model selection penalty (*γ* = 0.1). The network with the lowest EBIC score was then selected. The resulting network was characterized by a set of nodes (symptoms) and edges (non-zero partial correlations, or the most meaningful conditional dependencies).

**Figure 2:**
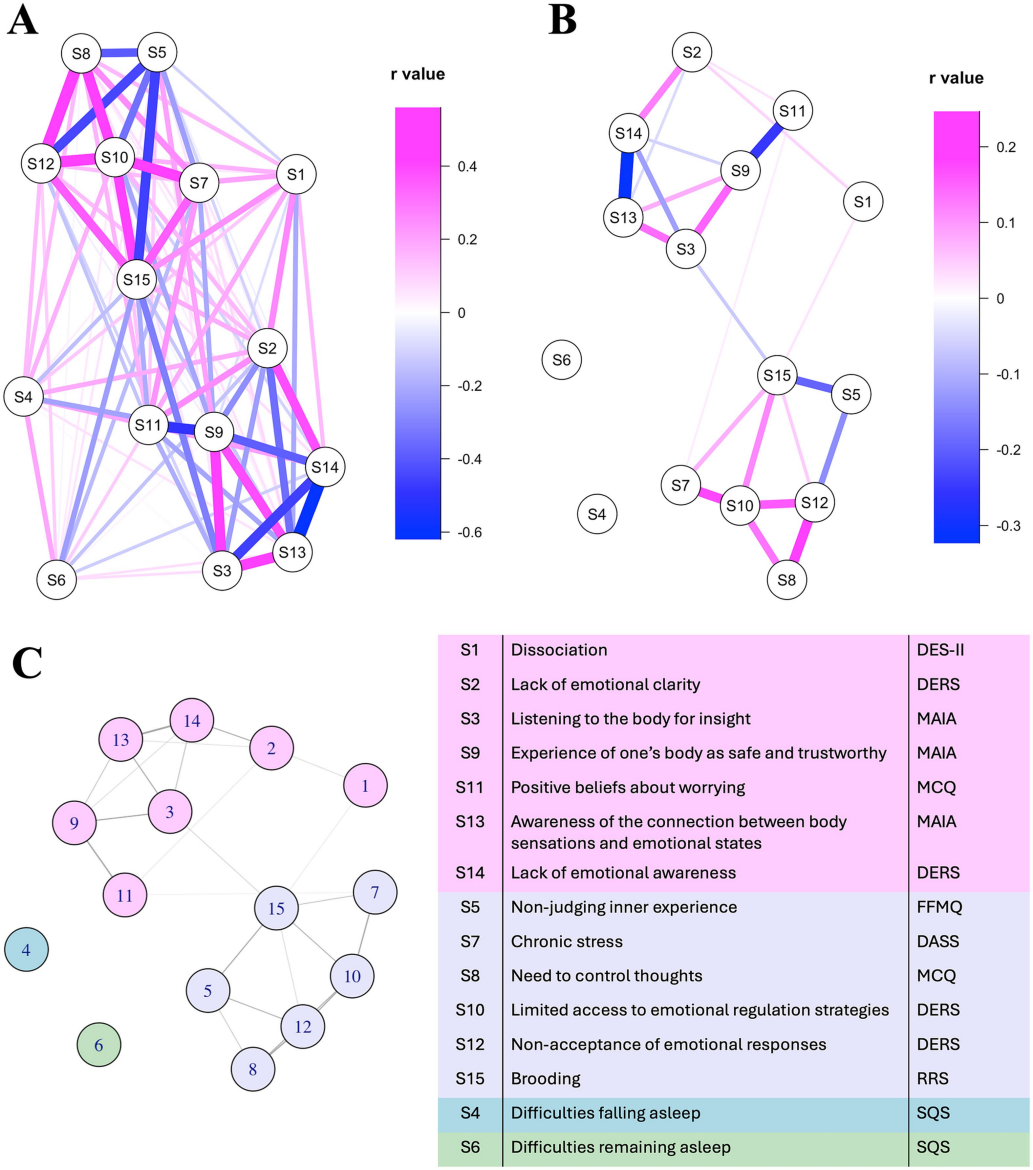
Interoceptive/emotional awareness symptoms and negative metacognitive symptoms form separate clusters in a network of brooding-associated symptoms. **(A)** Network using an unregularized correlation matrix. **(B)** Regularized sparse network of partial correlations. **(C)** Cluster assignment from the community detection algorithm.

For interpretability purposes, we computed two separate networks: one including the total MCQ score and one excluding it. The total MCQ is thought to be a general measure of dysfunctional metacognitive beliefs, with a higher score indicating greater dysfunction. The network, including the total MCQ score, demonstrated close relationships between the total MCQ score and other MCQ symptoms due to inherent multicollinearity, which may obscure other symptom relationships (Supplementary Figure 3). We therefore re-estimated the network after excluding the total MCQ score to aid with interpretability (Figure 2).

#### 2.2.3 Assessing edge weight stability and symptom clustering

We expected our estimated network to be unstable due to our limited sample size. Since the goal of this analysis was to generate hypotheses and identify candidate features for subsequent EEG analyses, rather than to derive precise population-level network parameters, we do not view edge-level instability as a severe limitation. Nonetheless, as it is best practice when reporting symptom networks (Epskamp et al., 2018; Epskamp and Fried, 2018), we assessed the stability of edge weights by constructing confidence intervals around the sample edge weights using nonparametric bootstrapping. Here, we resampled the data 5,000 times with replacement. To aid in interpreting patterns within high-level network structure, a community detection algorithm was used to identify symptom clusters using the *igraph* package in R (version 2.1.4) (Csárdi et al., 2025). The community detection algorithm performed short random walks from each node and computed how often two nodes co-occured in the same walk. If two nodes co-occured frequently, they were assigned to the same cluster.

### 2.3 EEG analyses

EEG preprocessing and source-localization were conducted using the *MNE* package in Python (v. 1.6.0) (Gramfort, 2013; Larson et al., 2025).

#### 2.3.1 Preprocessing

To remove high-frequency noise and low-frequency drift effects, we applied high (80 Hz) and low (1 Hz) pass windowed-sinc finite impulse response (FIR) filters. Next, we used a notch filter (defined in *MNE* as a symmetric FIR band-stop filter) to remove the electrical line noise artifact at 60 Hz. An independent components analysis (i.e, FastICA algorithm) was then used to identify eye blink and muscle movement related artifacts, with components removed based on visual inspection. On average, 4.7 out of 20 components were removed per participant, with components identified using both spectral and topographical features. The number of ICA components (20) was chosen based on the cumulative explained variance of the leading principal components which accounted for >95% of signal variance. Artifact related activity was therefore likely to be confined to these components. Data and power spectra were visually inspected after each component was removed until data were deemed sufficiently preprocessed for each participant.

#### 2.3.2 Source-localization

The data were then segmented into 2-s non-overlapping epochs prior to source localization. We applied the linearly constrained minimum variance (LCMV) beamforming technique to our preprocessed data to estimate source-level activity for regions of interest (ROIs) within frontocingulate-parietal-limbic circuits that are known to be involved in self-regulatory processes, rumination, and depression (Table 1). We selected the following ROIs *a priori*. In addition, we included areas known to play a role in attention/executive function (dorsolateral prefrontal cortex, or dlPFC) and memory (parahippocampal gyrus) as ruminative brooding is associated with executive function deficits (Koster et al., 2011; Singh et al., 2025; van Vugt et al., 2018; Yang et al., 2017), repetitive dwelling on past negative memories (Lyubomirsky et al., 1998), and overgeneral autobiographical memory retrieval (Sutherland and Bryant, 2007). The following ROIs were selected using the Destrieux cortical parcellation atlas (Destrieux et al., 2010): posterior cingulate cortex (PCC), anterior cingulate cortex (ACC), dlPFC, ventromedial prefrontal cortex (vmPFC), posterior parietal cortex (PPC), insula, somatosensory cortex (SSC), subcallosal cingulate (SCC), and parahippocampal gyrus (PHG). Destrieux labels, along with detailed justification for each ROI, are shown in Table 1.

After defining our ROIs, we used the LCMV beamformer to reconstruct source-localized time series (Van Veen et al., 1997) using modules available through the *MNE* Python package. Beamforming requires three major components: a covariance matrix, a forward model, and finally the LCMV filter. Forward models capture how source-level activity projects to electrodes on the scalp, and was constructed by combining a template brain (*fsaverage* from FreeSurfer with 5 mm spacing cortical source space), a “map” of electrode placement (the standard BIOSEMI 128-channel montage) and a model of how electrical activity passes through the inner skull, outer skull, and skin (a three-layer boundary element method, or BEM, model). The covariance matrix captures, for each pair of electrodes *i, j*, how the activity from electrode *i* covaries with the activity from electrode *j*. LCMV then combines the inverse covariance matrix with the forward model to create spatial filters for each target source, only allowing activity from a specific brain region to pass through while attenuating (i.e., minimizing total variance) signals from all other sources. Given that the covariance matrices were computed for resting state EEG, both the noise and data covariance matrices were identical. LCMV spatial filters were applied to each epoch to obtain time-resolved source estimates. Since each ROI has a set of sources studded at 5 mm intervals across its cortical region (because of the *fsaverage* template brain), with each source’s dipole perpendicular to the cortical surface, there may be some dipoles that are flipped relative to others by virtue of the curvature of the cortex. To obtain an averaged time series for each ROI, these sources therefore needed to be averaged while accounting for signal polarity. To accomplish this, we applied the *mean_flip* method in *MNE* to each epoch. This method computed the average dipole orientation within the ROI and flipped the sign of time series from sources that were oriented in the opposite direction. Epochs were then concatenated end-to-end to create a time series spanning the entire resting-state condition.

#### 2.3.3 Cross-frequency coupling

To compute phase-amplitude coupling strength (PAC), we used the *pactools* (v. 0.3.1) Python library. We computed PAC using the driven auto-regressive (DAR) model (Tour et al., 2017), which models high-frequency components (i.e., gamma and beta) as an autoregressive process whose coefficients are modulated by low-frequency (i.e., delta, theta and beta) phase. This method is more sensitive to true coupling vs. noise/artifact related effects, and is able to model directionality of coupling (i.e., whether low-frequency phase *modulates* high-frequency amplitude), compared to other standard methods (e.g., modulation index; Tort et al., 2010). PAC between theta (4–8 Hz) and gamma (30–80 Hz); beta (20–30 Hz) and gamma; and delta (2–4 Hz) and beta was computed for each ROI pair to create connectivity matrices for each participant. Instantaneous phase and amplitude sources were extracted from each ROI by applying Hilbert transformations and combined before applying the DAR method to facilitate the study of directional amplitude modulation and connectivity.

### 2.4 Assessing relationships between subscale measures and EEG metrics

#### 2.4.1 Partial least squares correlation (PLS-C)

To assess relationships between the subscale measures and PAC strength, we used partial least squares correlation (PLS-C) (Krishnan et al., 2011). PLS-C is a multivariate statistical method that extracts latent variables capturing patterns of maximal covariance between EEG features and subscale scores (i.e., components that maximize shared variance between the two feature sets) (Helland, 1990). Unlike univariate correlational analyses that test each ROI-symptom pair independently, PLS-C identifies abstract, higher-order patterns of co-variation that link sets of brain features (e.g., CFC between two brain regions) with sets of symptom variables (e.g., rumination, mindfulness, interoception, emotion regulation, etc). Therefore, PLS-C results are not interpreted as individual ROI-symptom correlations, but as emergent dimensions reflecting shared variance across many interrelated measures (McIntosh and Mišić, 2013).

PLS-C analyses were conducted in Python using the *sklearn.cross_decomposition* module from the *scikit-learn* package. To reduce the dimensionality of our set of independent variables, we selected five symptoms that we deemed were representative of the symptom clusters identified in our symptom network analysis. We assessed the statistical significance of each of the five latent variables using a permutation test, which computed *p-*values for each dimension by comparing the original model to 3,000 PLS-C models fit to randomly shuffled data (Moore, 1999). We used a Bonferroni-corrected statistical significance threshold of *α* = 0.01 to account for the five latent dimensions we evaluated.

## 3 Results

### 3.1 Levels of depression, anxiety and stress in the sample

The distributions of DASS-21 subscale scores are presented in Supplementary Figure 1, with a summary presented in Tables 2, 3. The majority of our participants were classified as within the “normal” range based on their DASS-21 depression subscale scores (54.2%, Table 2), with the remainder classified as exhibiting mild to severe depression symptoms (45.8%, Table 2). In contrast, the majority of our participants exhibited mild to extremely severe anxiety (62.5%, Table 2) and mild to severe perceived chronic stress (62.5%, Table 2). We then assessed degrees of depression, anxiety and perceived chronic stress in our sub-sample of *N =* 31 participants used for the subsequent EEG analyses. Results are presented in Table 3 below. As with the complete sample, the majority of our participants exhibited low levels of depression, consistent with the “normal” classification (58.1%, Table 3), and exhibited elevated levels of anxiety and stress, consistent with mild to extremely severe (64.5%, Table 3) and mild to severe (58.1%, Table 3) classifications, respectively. Therefore, these results indicate that a considerable proportion of our participants experienced elevated levels of depression, anxiety, and stress symptoms.

**Table 2:** Degree of depression, anxiety and perceived chronic stress levels in the total sample used for symptom network analyses (*N =* 48).

Severity level	Depression subscale		Anxiety subscale		Stress subscale	
	*n*	%	*n*	%	*n*	%
Normal	26	54.2	18	37.5d	18	37.5
Mild	6	12.5	3	6.2	17	35.4
Moderate	12	25	15	31.2	10	20.8
Severe	4	8.3	4	8.3	3	6.2
Extremely severe	0	0	8	16.7	0	0

**Table 3:** Degree of depression, anxiety and perceived chronic stress levels in the sub-sample used for symptom network analyses (*N =* 31).

Severity level	Depression subscale		Anxiety subscale		Stress subscale	
	*n*	%	*n*	%	*n*	%
Normal	18	58.1	11	35.5	13	41.9
Mild	4	12.9	3	9.7	11	35.5
Moderate	7	22.6	7	22.6	5	16.1
Severe	2	6.5	4	12.9	2	6.5
Extremely severe	0	0	6	19.4	0	0

### 3.2 Symptoms most relevant to ruminative brooding

Our Elastic Net regression identified the following 15 symptoms with the largest absolute coefficient magnitudes in relation to brooding (Supplementary Figure 2): trusting inner experience (MAIA-2), dissociation (DES-II), nonaccepting of emotions (DERS), lack of emotional regulation strategies (DERS), lack of emotional clarity (DERS), chronic stress (DASS-21), difficulty falling asleep (SQS), difficulty maintaining sleep (SQS), positive beliefs about worrying (MCQ), need to control thoughts (MCQ), general disturbances in metacognitive function (total MCQ), lack of emotional awareness (MAIA-2), nonjudging inner experience (FFMQ-39), and listening to the body for insight (MAIA-2). We used these symptoms for subsequent network analyses.

We must preface our symptom network results with a note that our edge weights were deemed unstable, likely due to our small sample size (Supplementary Figure 4); therefore, edge weights (Supplementary Table 1) should not be over-interpreted and should be viewed as hypothesis-generating and exploratory, as they need validation in a much larger sample. Nonetheless, the higher-level pattern of symptom clustering still reveals general relationships between symptoms; we therefore used these results to inform our subsequent EEG analyses. Our symptom network analysis and clustering algorithm revealed an exploratory trend of two clusters of symptoms relevant to ruminative brooding (see Figure 2B), with brooding included in one cluster but not the other. The first cluster, including brooding, contained negative symptoms relating to emotional dysregulation, self-judgement, chronic stress, a rejection of one’s emotional experiences, and a need to control one’s thoughts. The second cluster, on the other hand, contained symptoms relevant to emotional and interoceptive awareness. Additionally, there were three “bridging” associations between these two clusters, the strongest of which was a negative association between brooding and the utilization of interoceptive cues (i.e., “listening to the body for insight”). Notably, cluster assignment remained consistent after including the total MCQ score (Supplementary Figure 3).

Based on our symptom network results, we chose a subset of symptoms that were (1) most strongly related to brooding, and (2) representative of both clusters, for the PLS-C analysis. To reduce the dimensionality of the symptom dataset, we chose to select 5 symptoms for this next stage of analysis. First, we selected a set of candidate symptoms based on the largest absolute edge weights associated with either brooding or the bridging symptom “listening to the body for insight” (i.e., *bodylisten_maia*). The candidate symptoms associated with brooding included “limited access to emotional regulation strategies” (DERS), “perceived chronic stress” (DASS-21), and “nonjudging inner experiences” (FFMQ-39). Candidate symptoms associated with “listening to the body for insight” included “experience of one’s body as safe and trustworthy” (MAIA-2), “awareness of the connection between body sensations and emotional states” (MAIA-2), and “lack of emotional awareness” (DERS). We then evaluated nonparametric bootstrap estimates for these candidate edges (Supplementary Figure 4, and Supplementary Table 2). For each edge, we examined the bootstrap mean and confidence interval to assess relative stability. Although all percentile confidence intervals included zero, candidate edges varied meaningfully in magnitude and relative stability. Final symptom selection prioritized edges that a) ranked among the highest in absolute magnitude and b) closer correspondence between sample and bootstrap mean estimates relative to other candidate edges. Using these criteria, we therefore selected the following symptoms from cluster one: brooding (RRS), “limited access to emotional regulation strategies” (DERS), and “nonjudging inner experience” (FFMQ-39). From cluster two, we selected “listening to the body for insight” (the only symptom related to brooding, i.e., the strongest “bridging” symptom) and “experience of one’s body as safe and trustworthy,” both from the MAIA-2.

### 3.3 Neural correlates of key symptoms related to brooding

**Figure 3:**
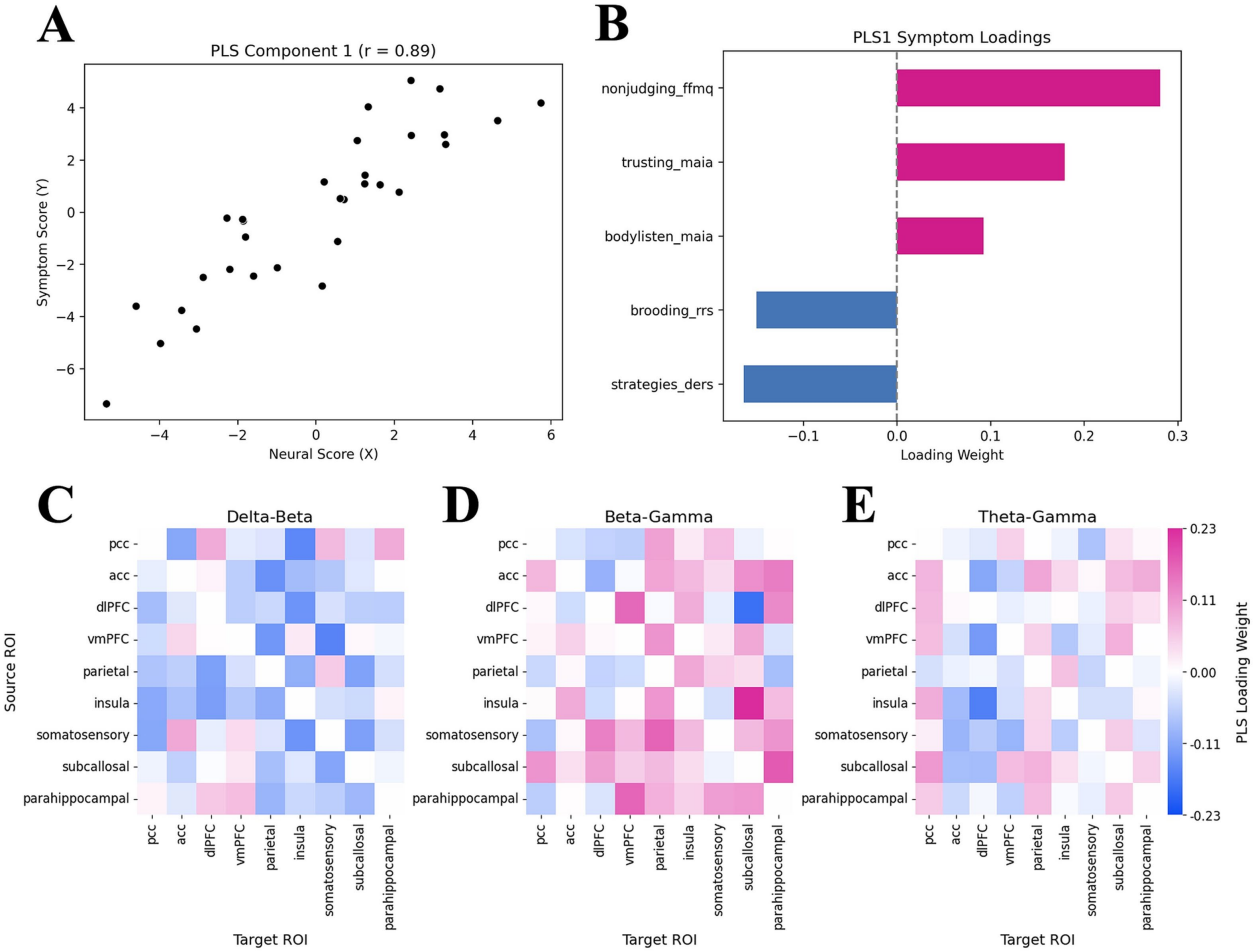
Partial least squares latent dimension 1 results, mapping core rumination symptoms to EEG cross-frequency coupling metrics. **(A)** PLS-C component 1 correlation (*r* = 0.89, *p* = 0.006 computed from permutation test), **(B)** Symptom loadings for first PLS-C dimension. Positive scores in the first dimension represent higher levels of mindfulness, while negative scores represent ruminative brooding and emotional dysregulation. **(C–E)** Heatmaps for cross-frequency coupling align with symptom loadings. Positive values (pink) align with mindfulness traits, while negative values (blue) align with ruminative brooding/emotional dysregulation. Symptom dimensions are as follows: nonjudging_ffmq = “non-judging inner experiences” subscale of the FFMQ-39; trusting_maia = “experience of one’s body as safe and trustworthy” subscale of the MAIA-2; bodylisten_maia = “listening to the body for insight” subscale of the MAIA-2; brooding_rrs = “brooding” subscale of the RRS; strategies_ders = “limited access to emotional regulation strategies” subscale of the DERS.

Only the first dimension of the PLS-C model was found to be statistically significant based upon our permutation test (r = 0.89, *p* = 0.006), meaning that the first latent variable captured a significant pattern of co-variation between the symptom and neural data. PLS-C weightings are plotted in Figure 3B, with associated heatmaps, representing cross-frequency coupling strength, shown in Figures 3C–E. The cross-frequency coupling pathways with the top 10 highest magnitude weights are also shown in Table 4 to aid with interpreting the high-level pattern of co-variation. Brooding and emotional dysregulation, represented by negative loading weights, were mostly associated with delta-beta CFC, while mindfulness traits, represented by positive loading weights, were mostly associated with beta-gamma CFC (with the exception of dlPFC→SCC, which had a negative loading aligning with brooding and emotional dysregulation). Theta-gamma CFC was generally associated with all symptom dimensions (Figure 3E), with the highest theta-gamma weight aligning with brooding and emotional dysregulation (Table 4). This diverging pattern of CFC signatures, linked to anticorrelated constructs such as emotional dysregulation/brooding and mindfulness, is consistent with prior literature: delta-beta CFC has been identified as a marker of emotional dysregulation (Myruski et al., 2022; Poppelaars et al., 2021), whereas beta-gamma coupling has been associated with mindfulness (Duda et al., 2024; Lomas et al., 2015; Ng et al., 2021). Since these features were entered into a multivariate covariance model, these associations should be interpreted as statistical co-variation rather than causal effects at the group level.

**Table 4:** Top 10 Neural pathway loading weights for the first latent dimension.

Oscillatory frequencies	ROIs	Loading weight	Symptom dimension
Theta-Gamma	Insula → dlPFC	−0.164	Brooding & ED
Delta-Beta	vmPFC → SSC	−0.157	Brooding & ED
	PCC → Insula	−0.153	Brooding & ED
	ACC → Parietal	−0.140	Brooding & ED
Beta-Gamma	dlPFC → SCC	−0.183	Brooding & ED
	dlPFC → vmPFC	0.160	Mindfulness
	Parahippocampal → vmPFC	0.163	Mindfulness
	SSC → Parietal	0.165	Mindfulness
	SCC → Parahippocampal	0.175	Mindfulness
	Insula → SCC	0.226	Mindfulness

ACC, anterior cingulate cortex; dlPFC, dorsolateral prefrontal cortex; ED, emotional dysregulation; PCC, posterior cingulate cortex; SCC, subcallosal cingulate cortex; SSC, somatosensory cortex; vmPFC, ventromedial prefrontal cortex.

**Figure 4:**
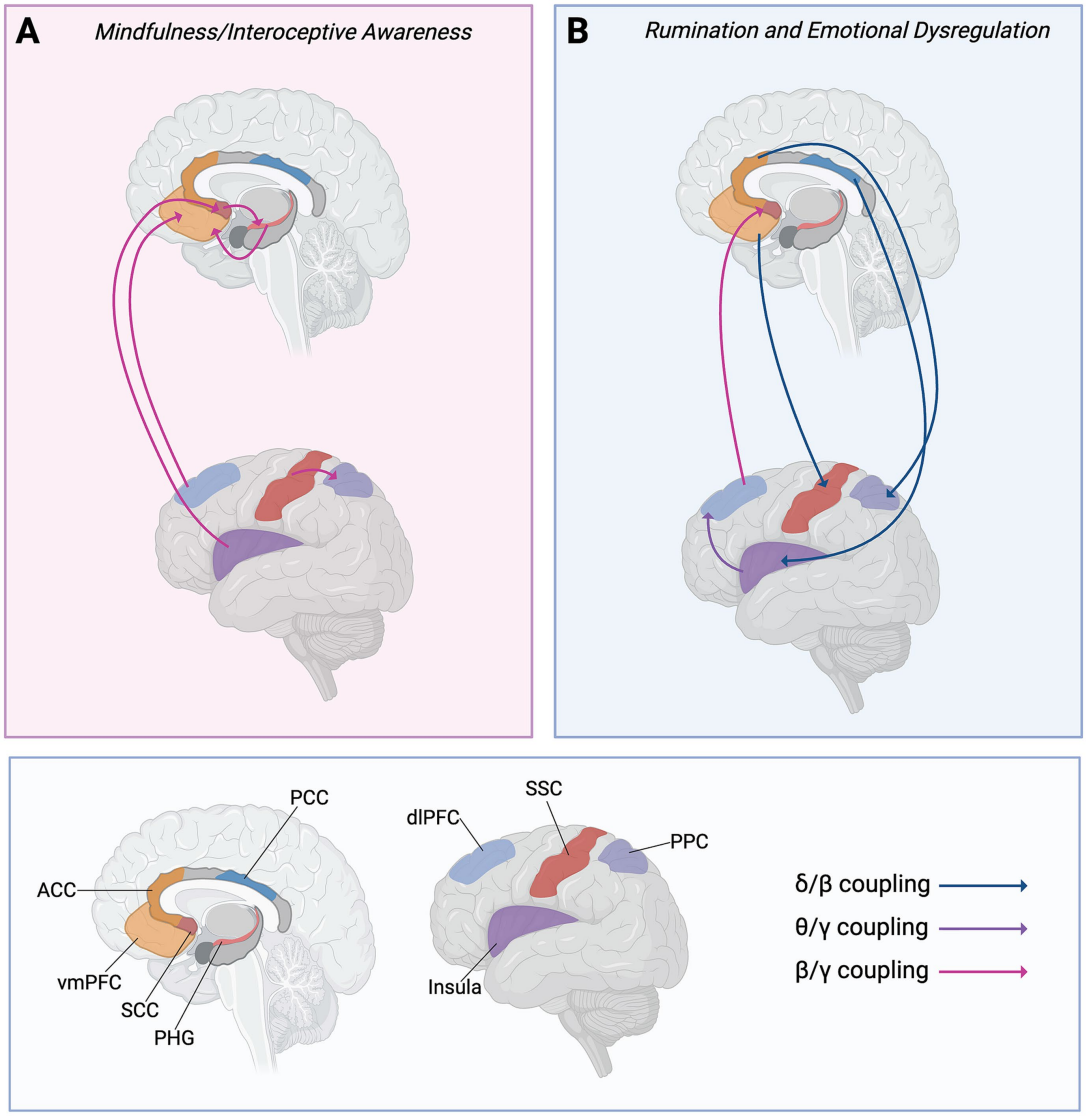
Circuit diagram of the top 10 neural pathway loading weights for the first latent dimension, corresponding with Table 4. **(A)** Cross-frequency coupling patterns co-varying with mindfulness/interoceptive awareness. **(B)** Cross-frequency coupling patterns co-varying with rumination/emotion dysregulation. Abbreviations: ACC = anterior cingulate cortex; dlPFC = dorsolateral prefrontal cortex; PCC = posterior cingulate cortex; PHG = parahippocampal gyrus; PPC = posterior parietal cortex; SCC = subcallosal cingulate cortex; SSC = somatosensory cortex; vmPFC = ventromedial prefrontal cortex.

CFC features that co-varied with symptom dimensions are mapped onto neural pathways in Figure 4. Importantly, our measure of CFC captures directed modulation between two regions. Here, it appears that brooding and emotional dysregulation were associated with a distributed pattern of prefrontal (vmPFC and dlPFC) and cingulate (ACC, PCC) coupling directed towards regions involved in interoceptive and self referential processing (somatosensory cortex, insula, posterior parietal cortex, subcallosal cingulate), with the exception of insula-to-dlPFC theta-gamma cross-frequency coupling (Figure 4). On the other hand, our data also show that the mindfulness dimension co-varied with CFC *within* these circuits (i.e., prefrontal, self-referential, emotional) as opposed to *between* them (Figure 4). Indeed, the dlPFC, vmPFC and parahippocampal gyrus appear to form one circuit, possibly modulated by subcallosal cingulate and the insula (Figure 4). The pattern of directed coupling from the somatosensory cortex to the posterior parietal cortex may represent the integration of interoceptive cues with higher-level self-referential processes.

## 4 Discussion

The present study explored how ruminative brooding relates to mindfulness/interoceptive awareness, emotion regulation and metacognitive beliefs, aiming to identify symptoms most strongly linked to brooding and to guide EEG analyses of CFC-based neural networks. We hypothesized that brooding would be associated with maladaptive metacognitive beliefs and low mindfulness/interoceptive awareness. Consistent with this hypothesis, our symptom cluster analyses revealed trends suggesting that brooding was clustered with chronic stress, poor emotion regulation, self-judgement and non-acceptance of emotional experiences, rather than with mindfulness/interoceptive awareness. The second symptom network cluster included interoceptive awareness and mindfulness dimensions, with the two clusters bridged by a negative association between brooding and “listening to the body for insight” (Figure 2). At the neural level, we hypothesized that brooding and mindfulness would engage overlapping circuits but differ in the *frequency* with which these regions synchronized, as well as in terms of which regions functioned as “modulators” vs. “processors.” Our measure of phase amplitude coupling, computed using the DAR method, enabled the analysis of *directional* CFC strength. The brooding/emotion dysregulation dimension co-varied with patterns of delta-beta and theta-gamma CFC and patterns, pointing towards fronto-cingulate modulation of the subcallosal cingulate, posterior parietal cortex and insula (Figures 3, 4); this could potentially represent top-down control and/or maladaptive appraisal of emotional and interoceptive cues. In contrast, mindfulness/interoceptive awareness traits (i.e., non-judgment of inner experience, listening to the body for insight, and bodily trust) co-varied with patterns of beta-gamma CFC mostly within affect/interoception-related regions (Figure 3 and Table 4). These mindfulness-related neural signatures appeared to reflect *within-system* synchrony, such that affect/interoception-related regions (subcallosal cingulate, parahippocampal gyrus, insula, posterior parietal, and somatosensory cortices) synchronized locally (Figure 4). Together, these exploratory results suggest that brooding dimensions co-varied with directed CFC from prefrontal and cingulate regions to affective and interoceptive regions, while mindfulness dimensions co-varied with patterns representing higher-frequency coordination *within* affective/interoceptive circuits. These patterns point towards a general resistance to emotional processes in brooding and emotion dysregulation, and the opposite pattern in mindfulness. This study therefore presents an integrative approach to investigating how factors that combine both top-down and bottom-up processes are related to brooding, and how these relationships are reflected neurally.

### 4.1 Brooding and emotional dysregulation

Ruminative brooding is classically thought to arise as a maladaptive emotional regulation process (Nolen-Hoeksema, 1991) and is perpetuated by an individual’s maladaptive metacognitive beliefs pertaining to their ruminative brooding (Papageorgiou and Wells, 2001; Wells and Matthews, 1996). Consistent with this, we observed the trend from our symptom network analysis that brooding was more closely linked to chronic stress, a lack of access to emotional regulation strategies, self-judgment, a need to control thoughts, and rejection of emotional responses, than to mindfulness and interoceptive awareness dimensions (Figure 2C). Our results suggest that brooding is associated with a general resistance to one’s internal experiences. Interoceptive cues may therefore be processed in a maladaptive way in our sample, with metacognitive beliefs leading to the rejection, judgement and distrust of internal experiences (Figure 2B). Indeed, brooding was weakly negatively associated with “listening to the body for insight”, which is also the bridging symptom between brooding and mindfulness symptom clusters (Figure 2B). This being said, brooding was also related to perceived chronic stress (Figure 2B). While acute stress exposure increases sensitivity to interoceptive cues, chronic stress exposure is thought to *reduce* sensitivity via regulatory allostasis (Schultchen et al., 2019). Regulatory allostasis refers to how the cumulative “wear and tear” of chronic stress leads to physiological dysregulation (Sterling, 2012). Future work should assess general sensitivity to interoceptive cues by including an appropriate validated measure (e.g., the bodily awareness questionnaire; Shields et al., 1989). Although these theoretical perspectives emphasize different mechanisms, our exploratory results, along with previous literature, suggest that they are not mutually exclusive but instead reflect an interconnected system in which metacognition, emotion, interoception, and stress regulation dynamically interact.

Aligning with the symptom clustering results, the patterns of co-variation between EEG CFC and brooding/emotional dysregulation (i.e., “limited access to emotional regulation strategies”) may potentially represent both top-down modulatory control over emotional regulation/interoceptive awareness cues and somatosensory-cognitive integration. These connectivity patterns align with symptom relationships, indicating a brooding-related rejection or resistance towards emotions. Consistent with prior literature, we observed that delta-beta signatures co-varied across the majority of ROIs with the emotional dysregulation/brooding dimension (Knyazev et al., 2006; Myruski et al., 2022; Poppelaars et al., 2021). As individual ROI–ROI relationships from PLS-C are less reliable than the overall pattern of co-variation, we present the following interpretation tentatively and as a basis for future study.

Within the top five negative PLS-C loadings, cingulate regions appear to function as “modulators” in terms of CFC. Cingulate involvement may be informative given the functional subdivisions of the ACC/PCC. The ACC is divided into a rostral-ventral affective division, connected to limbic and subcortical regions, including the amygdala, periaqueductal gray, nucleus accumbens, hypothalamus, anterior insula, hippocampus, and orbitofrontal cortex, while the dorsal cognitive division is linked to lateral prefrontal, parietal, premotor and somatosensory-motor cortices (Bush et al., 2000; Devinsky et al., 1995). We speculate that our finding of ACC modulation of the PPC points towards engagement of the dorsal division, indicating a role for modulatory cognitive control rather than emotional responding. Such a process would be consistent with our symptom network analysis, and if validated in a larger sample, may reveal an association between brooding and resistance to emotions.

The pattern indicating PCC modulation of the insula is also noteworthy, as it may point towards maladaptive integration of self-referential and memory-related processes with interoceptive cues. The PCC is interconnected with the parahippocampal gyrus and entorhinal cortex (and thus the hippocampal memory system) (Kobayashi and Amaral, 2003; Leech and Sharp, 2014; Suzuki and Amaral, 1994). As a key node within the brain’s default mode network, the PCC is also implicated in autobiographical memory recall (Conway et al., 1999; Fink et al., 1996; Maddock et al., 2001) and self-referential processing such as rumination (Benschop et al., 2021; Berman et al., 2011). The insula is substantially involved in emotional processing and interoceptive awareness (Berntson and Khalsa, 2021; Gu et al., 2013; Khalsa et al., 2009; Menon and Uddin, 2010). It is also functionally parcellated into ventro-anterior and dorsal-posterior regions, with the anterior portion linked to the ACC and limbic regions, while the posterior portion is linked to the PCC and somatosensory cortex (Cauda et al., 2011). Synchrony between the PCC and insula may facilitate integration of self-referent and somatosensory/interoceptive cues, and has been associated with ruminative brooding; this previous work proposed that this relationship is indicative of *increased* bodily awareness (Li et al., 2022). Here, our results may be representing a rumination circuit that integrates self-referential processing and interoceptive cues (PCC → insula→dlPFC and the parallel vmPFC→somatosensory cortex pathway), however, the co-variation between this circuit’s activity patterns with the brooding and emotional dysregulation dimensions may reflect a *maladaptive* appraisal process. Considering the exploratory trends revealed in our symptom network analyses, individuals may intellectualize or rationalize their inner experience, deeming it untrustworthy and unhelpful (Figure 2B), which ultimately leads to the rejection of their emotions and interoceptive cues. This process may be occurring together with active ruminative brooding. Future work should examine the relationship between the sensitivity to and appraisal of interoceptive cues and rumination.

Alongside circuits implicated in maladaptive integration of emotion and interoceptive cues, our results are also consistent with findings of a well-established depression circuit that may similarly reflect maladaptive compensatory top-down control; this circuit includes the dlPFC and SCC (Benschop et al., 2022; Drevets et al., 2008; Hamani et al., 2011). One of the highest PLS-C CFC loadings was dlPFC-driven beta phase modulation of gamma amplitude in the SCC (Table 4), which may reflect a trend-level association between dlPFC-SCC CFC and brooding/emotion dysregulation. The SCC is a critical node in the neurobiology of depression (Drevets et al., 2008; Hamani et al., 2011), thought to process negative emotions (Vogt, 2014) through projections to autonomic regulatory centers, including the hypothalamus, amygdala and periaqueductal grey (Vogt and Vogt, 2009). The dlPFC→SCC pathway has been widely implicated in depression, with aberrant connectivity predicting major depressive disorder (Benschop et al., 2022) and treatment responsiveness (Cr et al., 2021; Fox et al., 2012; Ge et al., 2020; Liu et al., 2019; Tan et al., 2023). Since our sub-sample of participants exhibited considerable degrees of depressive, anxious and stress-related symptoms (Table 3), the heightened dlPFC→SCC connectivity we observed may be a valid marker of brooding, reflecting compensatory cognitive control over SCC-mediated negative affect at rest. As we have stated before, given the exploratory nature of this study, these individual ROI-ROI relationships should be investigated in further detail in a larger sample. In addition, task-based paradigms should be included to evaluate whether the observed CFC patterns truly reflect modulatory influence of the prefrontal regions (i.e., control and/or compensation) over regions involved in affective regulation.

### 4.2 Mindfulness and interoceptive awareness

The mindfulness/interoceptive awareness cluster in our symptom network demonstrates a mix of positive and negative symptom associations. Positively associated symptoms reflect adaptive integration and processing of interoceptive cues, and include listening to the body for insight, awareness of the connection between body sensations and emotional states, and experiencing the body as safe and trustworthy (Figure 2B). In addition, there are positive associations between maladaptive symptoms, such as lack of emotional clarity, lack of emotional awareness, dissociation and positive beliefs about worrying (Figure 2B). Together, this symptom cluster may represent processes that utilize interoceptive and emotional cues for self-regulation, which is the core of mindfulness.

In terms of neural processes, mindfulness dimensions more strongly co-varied with beta-gamma, and to some extent and theta-gamma CFC patterns, rather than delta-beta (Figure 3); this aligns with known neural correlates of mindfulness (Duda et al., 2024, p. 20). Within the top five positive loadings associated with mindfulness symptoms (Table 4), our results point toward circuits that perform somatosensory integration and emotional regulation (Figure 4). One pathway, consisting of the insula, SCC, and parahippocampal gyrus, appears to converge onto the vmPFC (Figure 4). The vmPFC may therefore function as the downstream “integrator” of emotional and somatosensory cues (Figure 4); the vmPFC is well-suited to facilitate appraisal of negative emotions, given its established role in regulation of negative emotion (Delgado et al., 2008; Johnstone et al., 2007; Urry et al., 2006) and self-referential processing (D’Argembeau, 2013). In a separate pathway, the somatosensory cortex appeared to modulate the PPC, which would also facilitate integration of sensory/bodily cues with self-referent processing and spatial awareness, aligning with anatomical projections between these regions (Leinonen, 1984). Together, this circuit may represent mindfulness as a process that involves integration of interoceptive, somatosensory and emotional cues with self-referential processes; this lies in direct contrast with the rumination/emotional dysregulation circuit, which may represent maladaptive appraisal and compensatory control over emotions and internal experiences. As with the CFC patterns that co-varied with emotional dysregulation/brooding dimension, these mindfulness-related CFC patterns and pathways must be validated in future work.

### 4.3 Clinical considerations

The exploratory results presented here point towards a link between brooding, self-judgement and a lack of emotional regulation strategies. Below, we present a discussion on the potential clinical pathways for future exploration that may be helpful for brooding, given the trends revealed by our exploratory symptom network analyses. Emotions can be regulated by either manipulating the evaluation of emotional triggers (e.g., thoughts, metacognitive beliefs) or by manipulating emotional responses (Hofmann and Asmundson, 2008). There are two classes of such therapies: cognitive therapies that incorporate mindfulness elements to promote appraisal of thoughts and emotions, and mindfulness-centred therapies that promote adaptive responses to emotions. Intuitively, mindfulness-based psychotherapies may be beneficial for ruminative brooding as they aim to reduce self-judgement and improve reflection and awareness of the relationship between mind and body. The therapies centred around mindfulness include mindfulness-based stress reduction (MBSR) (Kabat-Zinn, 1982) and mindfulness-based cognitive therapy (MBCT) (Segal et al., 2018). Both of these therapies effectively reduce anxiety and improve emotion regulation (Hofmann et al., 2010) with effect sizes comparable to that of cognitive-behavioural therapy (CBT) (Hofmann and Gómez, 2017). MBCT combines principles of cognitive therapy with increasing internal awareness to teach individuals how to disengage from repetitive negative thinking (Shahar et al., 2010), making this form of therapy effective at reducing rumination (Foroughi et al., 2020; Van Vugt et al., 2012) and effective for individuals with recurring depression (Ma and Teasdale, 2004).

Other therapies place a greater emphasis on cognitive reappraisal of thoughts that precede emotion, but may still incorporate mindfulness-based components. These include CBT (Boswell et al., 2014) and metacognitive therapy (MCT) (Capobianco and Nordahl, 2023). These two differ in their focus, with CBT centred on the thought *content* preceding emotion, while MCT is centred on the thought *processes* themselves. CBT teaches how thought content impacts emotion and subsequent behaviour, and encourages individuals to challenge maladaptive thought content by questioning the accuracy and relevance of those thoughts. Without reappraising negative thoughts and replacing them with neutral ones, subsequent negative moods may persist and reinforce ruminative thinking. In contrast to CBT, MCT is based on the self-regulatory executive function (S-REF) model, in which metacognitive beliefs create a “cognitive attentional syndrome”, referring to perseverative negative thinking (e.g., worry and ruminative brooding) (Wells and Matthews, 1996). MCT uses “detached mindfulness” to facilitate metacognitive modification, which involves placing the individual in an “observer” position followed by a therapist-guided discussion on a patient’s beliefs about their thoughts and how they relate to them, rather than focusing on thought content specifically and challenging validity. Both CBT and MCT are effective at reducing rumination and improving depressive and anxious symptoms (Abdollahi et al., 2021; Ballesio et al., 2018; Wells et al., 2012), despite modulating different mechanisms.

The next natural question is: since both cognitive- and mindfulness-centred therapies are effective for rumination, which one is better? Prior literature suggests that therapy selection may be dependent on symptom severity; acceptance and commitment therapy (ACT) (Hayes et al., 1999), a mindfulness-based therapy, is shown to be effective in reducing depressive symptoms acutely in those with mild depression, but may not be as effective in those with more severe symptoms (Bai et al., 2020). MBCT as an adjunct to standard psycho- and pharmacotherapy for chronic depression did not yield greater reduction in depressive symptoms compared to usual treatment alone, but did improve remission rates, rumination and self-compassion in those who completed the intervention (Cladder-Micus et al., 2018). Additionally, MBCT significantly decreased depression severity and improved treatment response rates in individuals with treatment-resistant depression, but did not yield improved remission rates compared to the control intervention (Eisendrath et al., 2016). As an alternative to MBCT, targeting metacognitive beliefs may yield more temporally durable symptom reductions; therapeutic reductions in rumination persisted after 1 year of MCT in individuals with treatment-resistant depression (Wells et al., 2012), highlighting the mechanistic importance of metacognitive beliefs in sustaining rumination. Therefore, in individuals with more severe depression, targeting metacognitive beliefs may be more beneficial for long-term management of rumination symptoms than promoting mindfulness strategies. However, mindfulness and cognitive therapy are both effective for reducing repetitive negative thinking and brooding by ultimately promoting adaptive appraisal of negative thoughts and metacognitive beliefs along with non-judgmental reactions to emotions. In the context of the exploratory analysis and results presented here in which we observed that the brooding-related symptom dimension co-varied with patterns of CFC consistent with prefrontal modulation of limbic and cingulate regions (Figure 3), we speculate that improving an individual’s ability to self-regulate emotions, by explicitly enabling the awareness and appraisal of emotional thoughts and bodily sensations, may lead to a reduction in ruminative brooding. Future work should therefore consider exploring the effects of combining *both* approaches for rumination, and evaluating the resulting impacts on CFC dynamics within frontal, cingulate and limbic regions to assess if there is a corresponding shift towards increased cross-regional integration (in alignment with a “mindfulness” based dimension) and reduced prefrontal modulation over regions involved with affective regulation (as we observed in brooding).

### 4.4 Strengths, limitations, and directions for future work

The present study has several notable strengths alongside important limitations. A key methodological strength is our novel integration of symptom network analysis with EEG, enabling us to generate hypotheses directly from symptom interdependencies and subsequently map these interdependencies to their underlying neural substrates in the same sample. To facilitate the linkage between these two datasets, we used PLS-C, which is particularly well-suited for cases with limited sample sizes, as it can handle multicollinearity and extract latent components that maximize the shared variance between the datasets. This makes PLS-C more feasible for our use case than traditional regression, as the number of neural features exceeds our number of participants. Despite these strengths, PLS-C is still sensitive to small sample sizes, which may inflate loadings. Although we incorporated permutation testing to evaluate the probability of observing a “true” (i.e., non-random) result, the stability of individual symptom-neural associations cannot be completely guaranteed. In a similar vein, we also reported instability in the symptom network edge weight estimates. According to best-practice guidelines, a network of 15 symptoms would require at least ~100 participants to support stability adequately (Epskamp et al., 2018; Epskamp and Fried, 2018). The clustering patterns and PLS-C results we reported must therefore be interpreted cautiously and validated in larger, more diverse samples (e.g., using the Max Planck Institut Leipzig Mind-Brain–Body dataset (Babayan et al., 2019)) using bootstrapping and stability analyses. The majority of our participants were female, and a substantial number of our participants (29%) did not report their sex/gender, limiting generalizability. The relatively high rate of missing demographic data may point toward excessive demands placed on our participants by the extensive questionnaire battery and lengthy EEG measures; indeed, the demographics questionnaire was administered after the study session. Furthermore, although our sample was “non-clinical” in the sense that participants did not report a formal psychiatric diagnosis, DASS-21 scores indicated elevated levels of depression, anxiety and stress symptoms in a subset of individuals. Importantly, “non-clinical” in this context refers to the absence of a confirmed diagnosis or treatment engagement, rather than the absence of psychological *distress*. Elevated DASS-21 scores are commonly observed in undergraduates, which may be attributable to increased academic pressures and major lifestyle changes (Beiter et al., 2015). Nonetheless, replication in clinically-diagnosed populations is necessary to evaluate whether the same symptom–neural associations generalize to individuals meeting formal diagnostic criteria for major depressive disorder. It remains possible that the elevated symptoms in our sample reflect subclinical distress rather than disorder-level psychopathology, which may differ in severity, chronicity, and underlying neurobiological mechanisms. Future work should therefore replicate these analyses in larger and more diverse samples.

Nonetheless, in support of the plausibility of our results, our use of high-density EEG (i.e., 128 leads) increased scalp-level sampling density relative to more commonly-used 32 and 64 lead systems. Greater sensory coverage supports source-level estimation and CFC computation. However, in the present study, source reconstruction was performed using a template brain, standard electrode positions, and a BEM forward model; while this approach is common in exploratory EEG research, source localization accuracy can be further improved by creating individual head models per participant (e.g., by combining magnetic resonance imaging and/or head digitization data), which we were not able to include due to resource constraints. This being said, the CFC-based circuits we presented align with previous findings in the literature regarding the neurobiology of mindfulness (Duda et al., 2024), rumination (Benschop et al., 2021; Ferdek et al., 2016), and signatures of emotional dysregulation (Myruski et al., 2022; Poppelaars et al., 2021). In addition, future work should also consider expanding the suite of neural signatures we included here. Our analyses combined both hemispheres, obscuring lateralization effects which have been reported in rumination before (Bocharov et al., 2021b; Ferdek et al., 2016). Alpha asymmetry, in particular, is a well-established marker of depression and brooding (Albu and Meagher, 2016; Berwian et al., 2024; Kaiser et al., 2018; Li et al., 2021), and may provide an important avenue for future investigation. Including hemispheric asymmetries and cross-hemisphere integration would thus further refine our understanding of the circuits underlying brooding, mindfulness, and interoceptive awareness.

Another important limitation to acknowledge is the risk of recursive interpretation; indeed, we selected ROIs based on previously-reported associations with rumination- and mindfulness-based constructs (Table 1), followed by interpreting findings within those regions in the context of those very same constructs. However, the primary aim of the present study was not to confirm regional activation-levels *per se*, but to examine oscillatory cross-frequency coupling patterns between regions and their relationships with multidimensional symptom dimensions spanning *both* adaptive (i.e., mindfulness) and maladaptive (i.e., brooding) processes. Therefore, the ROI selection constrained our search space, but our hypotheses and aims importantly were centered on connectivity dynamics and symptom variability, reducing the risk of circular confirmation. Accordingly, our findings should be interpreted as *consistent with* prior neurobiological models of rumination and self-regulatory processing. Future work employing whole-brain or data-driven parcellation approaches will be important for evaluating the generalizability of these circuit-level findings.

The present study also employed a 5-min eyes-closed resting-state EEG protocol, which is susceptible to fluctuations in vigilance. Even over shorter recording intervals, participants may transition toward drowsiness, which may influence oscillatory power and CFC metrics. Future studies may benefit from incorporating objective measures of vigilance (e.g., performance on the psychomotor vigilance task; Dinges and Powell, 1985; Dorrian et al., 2004) or alternating eyes-open and eyes-closed conditions to constrain arousal-related confounds. Furthermore, residual muscle-related contamination may remain despite our preprocessing and ICA-based artifact rejection protocol, which may impact PAC metrics involving high-frequency oscillations (i.e., gamma). Future studies may therefore also benefit from concurrent electromyographic recordings to further dissociate neural from artifactual contributions to high-frequency PAC.

To better capture dynamic relationships between stress exposure, appraisal processes, and brooding-related symptoms, future work should also consider adopting a longitudinal design. Such an approach would enable the study of how life stressors dynamically modulate the associations between mindfulness, emotion regulation and brooding-related symptoms. This would be particularly informative from the perspective of the allostatic regulation hypothesis, which frames diminished interoceptive awareness as a consequence of chronic stress exposure (Schultchen et al., 2019). Repeated EEG recordings would also enable tracking of how allostatic regulation shapes neural networks and oscillatory dynamics. Indeed, cortisol and noradrenaline receptors exist throughout the brain, notably within prefrontal, hippocampal and limbic circuits (Lenart-Bugla et al., 2022); these stress-related neuromodulators are known to influence neuroplasticity and neurogenesis (McEwen and Gianaros, 2011), which may ultimately impact interoception, executive function, emotional regulation and ruminative brooding.

## 5 Conclusion

We presented an exploratory analysis that integrated symptom network relationships with EEG-derived CFC neural circuits to examine how ruminative brooding, mindfulness, interoceptive awareness and metacognitive beliefs map onto neural circuits. Symptom clustering revealed that brooding aligned with emotional dysregulation and maladaptive metacognitive processes, while mindfulness and interoceptive awareness traits formed a separate cluster. At the neural level, brooding dimensions co-varied with patterns of low-frequency CFC, possibly representing compensatory top-down control and maladaptive appraisal of interoceptive cues. In contrast, mindfulness and interoceptive traits co-varied with higher-frequency beta-gamma and theta-gamma CFC *within* control, affective and interoceptive circuits. Future work should validate these exploratory trends in a larger sample to assess generalizability. Taken together, our findings lead us to propose that ruminative brooding is marked by a rejection of one’s emotions and inner experiences, which may also be reflected in the associated neural connectivity. These results may inform testable hypotheses for future research, and also the development of integrative neurocognitive theories of brooding.

## Data Availability

The raw data supporting the conclusions of this article will be made available by the authors, without undue reservation.
