# Supplementary material for: Frontocingulate-parietal-limbic circuits associated with both ruminative brooding and self-regulatory processes
Source: Front Hum Neurosci. 2026 Mar 13;20:1731382. doi: 10.3389/fnhum.2026.1731382 (PMC13021775; doi:10.3389/fnhum.2026.1731382)
Supplement: Supplementary file 1 [file Data_Sheet_1.docx]

# *Supplementary Materials for:*

# Frontocingulate-parietal-limbic circuits associated with both ruminative brooding and self-regulatory processes

**Short Title:** Mind-body circuits in brooding

Selena Singh [1], Vibooshitha Thusyanthan [1], Allison Mizzi [1, 2], Yarden Levy [1, 3], Isaac Kinley [4, 5], Saurabh Bhaskar Shaw [6], Suzanna Becker [1,*]

[1] Department of Psychology, Neuroscience and Behaviour; McMaster University, Hamilton, Ontario

[2] Nova Scotia Health Authority, Halifax, Nova Scotia

[3] Kaplan and Levitt Psychologists, Hamilton, Ontario

[4] Rotman Research Institute, Baycrest Academy for Research and Education, Toronto, Ontario, Canada

[5] Data Sciences Institute, University of Toronto, Toronto, Ontario, Canada

[6] Western University, London, Ontario

* *correspondence: beckers@mcmaster.ca*

# 1. Supplementary Results


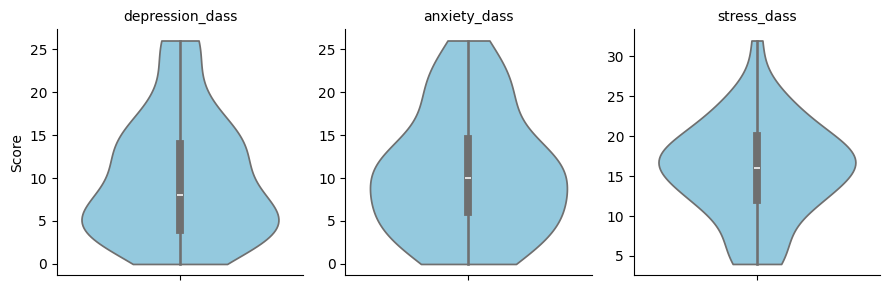


*Figure 1: Score distribution for depression, anxiety and stress subscales of the DASS.*

## 1.1. Elastic net features


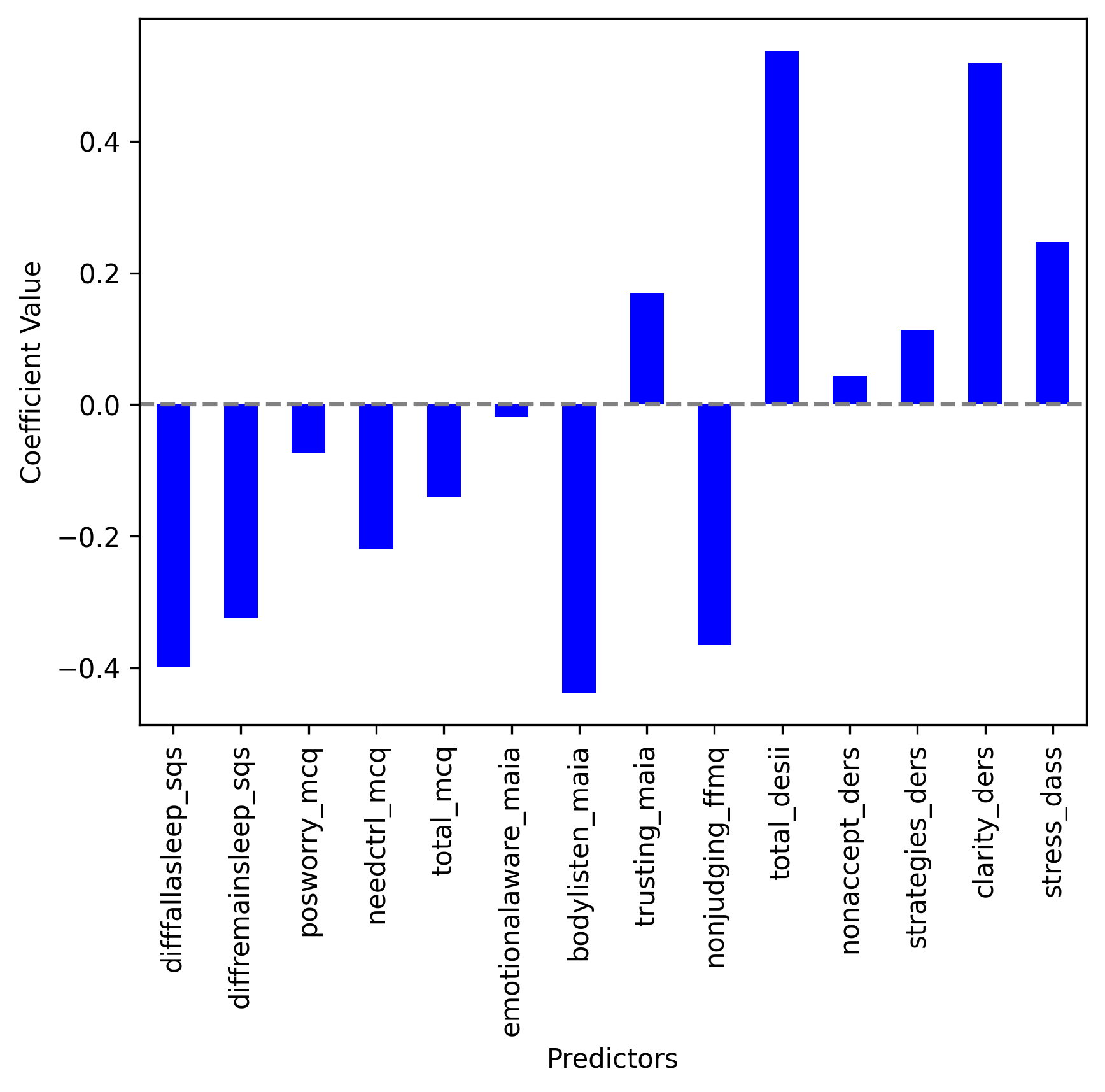


*Figure 2: Top 15 features correlated with brooding selected from elastic net regression.*

## 1.2. Network including total MCQ


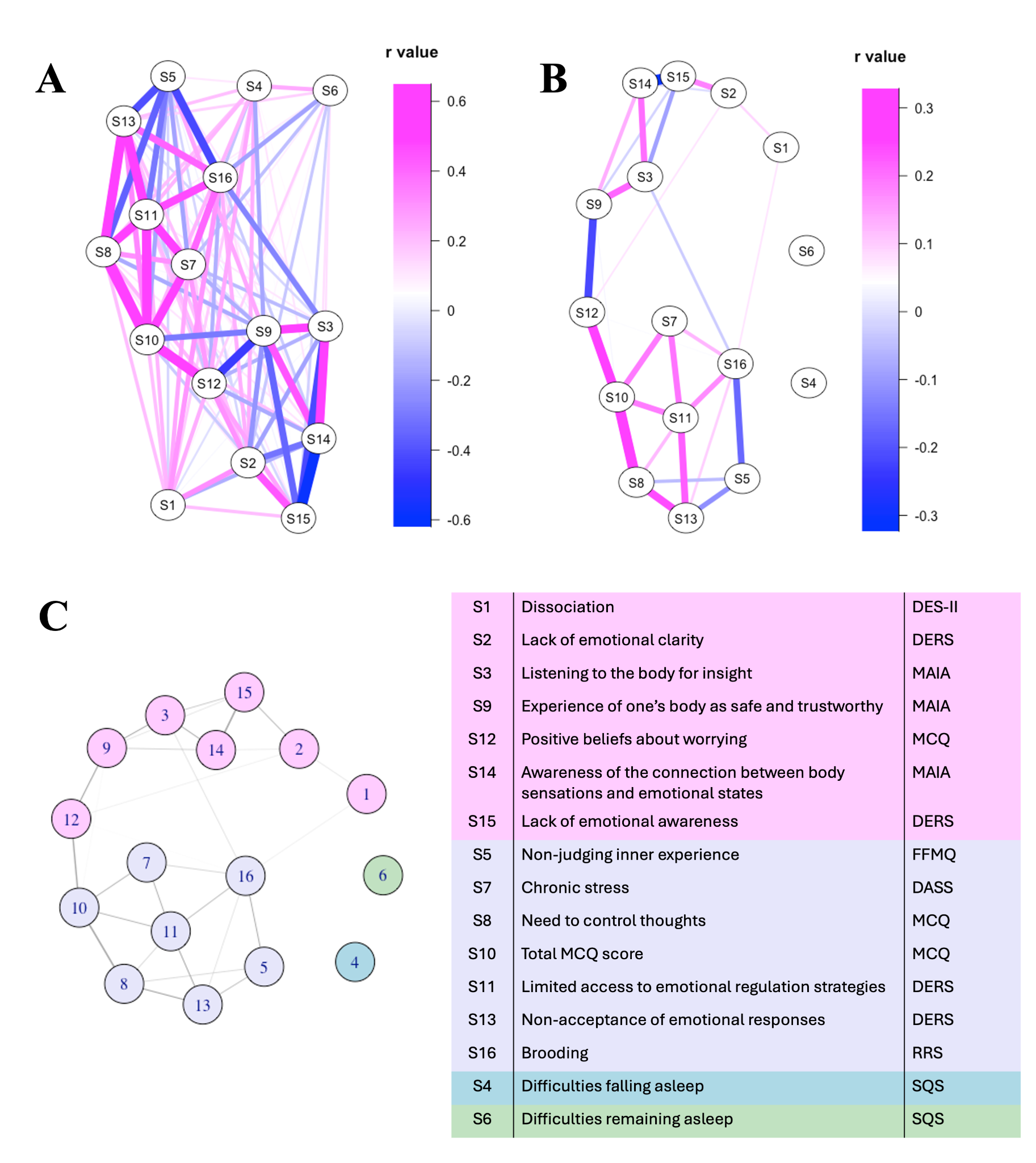


*Figure 3: Symptom network analysis including total MCQ score. Clustering behaviour is consistent with symptom network presented in the main text. A) Network using unregularized correlation matrix. B) Regularized sparse network of partial correlations. C) Cluster assignment from community detection algorithm.*

Table 1: Edge weights from symptom network

| **Node 1** | **Node 2** | **Edge Weight** |
| --- | --- | --- |
| total_desii | clarity_ders | 0.053 |
| total_desii | bodylisten_maia | 0.000 |
| clarity_ders | bodylisten_maia | 0.000 |
| total_desii | difffallasleep_sqs | 0.000 |
| clarity_ders | difffallasleep_sqs | 0.000 |
| bodylisten_maia | difffallasleep_sqs | 0.000 |
| total_desii | nonjudging_ffmq | 0.000 |
| clarity_ders | nonjudging_ffmq | 0.000 |
| bodylisten_maia | nonjudging_ffmq | 0.000 |
| difffallasleep_sqs | nonjudging_ffmq | 0.000 |
| total_desii | diffremainsleep_sqs | 0.000 |
| clarity_ders | diffremainsleep_sqs | 0.000 |
| bodylisten_maia | diffremainsleep_sqs | 0.000 |
| difffallasleep_sqs | diffremainsleep_sqs | 0.000 |
| nonjudging_ffmq | diffremainsleep_sqs | 0.000 |
| total_desii | stress_dass | 0.000 |
| clarity_ders | stress_dass | 0.000 |
| bodylisten_maia | stress_dass | 0.000 |
| difffallasleep_sqs | stress_dass | 0.000 |
| nonjudging_ffmq | stress_dass | 0.000 |
| diffremainsleep_sqs | stress_dass | 0.000 |
| total_desii | needctrl_mcq | 0.000 |
| clarity_ders | needctrl_mcq | 0.000 |
| bodylisten_maia | needctrl_mcq | 0.000 |
| difffallasleep_sqs | needctrl_mcq | 0.000 |
| nonjudging_ffmq | needctrl_mcq | -0.093 |
| diffremainsleep_sqs | needctrl_mcq | 0.000 |
| stress_dass | needctrl_mcq | 0.000 |
| total_desii | trusting_maia | 0.000 |
| clarity_ders | trusting_maia | 0.000 |
| bodylisten_maia | trusting_maia | 0.196 |
| difffallasleep_sqs | trusting_maia | 0.000 |
| nonjudging_ffmq | trusting_maia | 0.000 |
| diffremainsleep_sqs | trusting_maia | 0.000 |
| stress_dass | trusting_maia | 0.000 |
| needctrl_mcq | trusting_maia | 0.000 |
| total_desii | strategies_ders | 0.000 |
| clarity_ders | strategies_ders | 0.000 |
| bodylisten_maia | strategies_ders | 0.000 |
| difffallasleep_sqs | strategies_ders | 0.000 |
| nonjudging_ffmq | strategies_ders | 0.000 |
| diffremainsleep_sqs | strategies_ders | 0.000 |
| stress_dass | strategies_ders | 0.232 |
| needctrl_mcq | strategies_ders | 0.175 |
| trusting_maia | strategies_ders | 0.000 |
| total_desii | posworry_mcq | 0.000 |
| clarity_ders | posworry_mcq | 0.035 |
| bodylisten_maia | posworry_mcq | 0.000 |
| difffallasleep_sqs | posworry_mcq | 0.000 |
| nonjudging_ffmq | posworry_mcq | 0.000 |
| diffremainsleep_sqs | posworry_mcq | 0.000 |
| stress_dass | posworry_mcq | 0.022 |
| needctrl_mcq | posworry_mcq | 0.000 |
| trusting_maia | posworry_mcq | -0.252 |
| strategies_ders | posworry_mcq | 0.000 |
| total_desii | nonaccept_ders | 0.000 |
| clarity_ders | nonaccept_ders | 0.000 |
| bodylisten_maia | nonaccept_ders | 0.000 |
| difffallasleep_sqs | nonaccept_ders | 0.000 |
| nonjudging_ffmq | nonaccept_ders | -0.143 |
| diffremainsleep_sqs | nonaccept_ders | 0.000 |
| stress_dass | nonaccept_ders | 0.000 |
| needctrl_mcq | nonaccept_ders | 0.245 |
| trusting_maia | nonaccept_ders | 0.000 |
| strategies_ders | nonaccept_ders | 0.208 |
| posworry_mcq | nonaccept_ders | 0.000 |
| total_desii | emotionalaware_maia | 0.000 |
| clarity_ders | emotionalaware_maia | -0.045 |
| bodylisten_maia | emotionalaware_maia | 0.186 |
| difffallasleep_sqs | emotionalaware_maia | 0.000 |
| nonjudging_ffmq | emotionalaware_maia | 0.000 |
| diffremainsleep_sqs | emotionalaware_maia | 0.000 |
| stress_dass | emotionalaware_maia | 0.000 |
| needctrl_mcq | emotionalaware_maia | 0.000 |
| trusting_maia | emotionalaware_maia | 0.104 |
| strategies_ders | emotionalaware_maia | 0.000 |
| posworry_mcq | emotionalaware_maia | 0.000 |
| nonaccept_ders | emotionalaware_maia | 0.000 |
| total_desii | awareness_ders | 0.000 |
| clarity_ders | awareness_ders | 0.162 |
| bodylisten_maia | awareness_ders | -0.120 |
| difffallasleep_sqs | awareness_ders | 0.000 |
| nonjudging_ffmq | awareness_ders | 0.000 |
| diffremainsleep_sqs | awareness_ders | 0.000 |
| stress_dass | awareness_ders | 0.000 |
| needctrl_mcq | awareness_ders | 0.000 |
| trusting_maia | awareness_ders | -0.056 |
| strategies_ders | awareness_ders | 0.000 |
| posworry_mcq | awareness_ders | 0.000 |
| nonaccept_ders | awareness_ders | 0.000 |
| emotionalaware_maia | awareness_ders | -0.322 |
| total_desii | brooding_rrs | 0.033 |
| clarity_ders | brooding_rrs | 0.000 |
| bodylisten_maia | brooding_rrs | -0.068 |
| difffallasleep_sqs | brooding_rrs | 0.000 |
| nonjudging_ffmq | brooding_rrs | -0.194 |
| diffremainsleep_sqs | brooding_rrs | 0.000 |
| stress_dass | brooding_rrs | 0.098 |
| needctrl_mcq | brooding_rrs | 0.000 |
| trusting_maia | brooding_rrs | 0.000 |
| strategies_ders | brooding_rrs | 0.150 |
| posworry_mcq | brooding_rrs | 0.000 |
| nonaccept_ders | brooding_rrs | 0.063 |
| emotionalaware_maia | brooding_rrs | 0.000 |
| awareness_ders | brooding_rrs | 0.000 |

## 1.3. Edge-weight stability


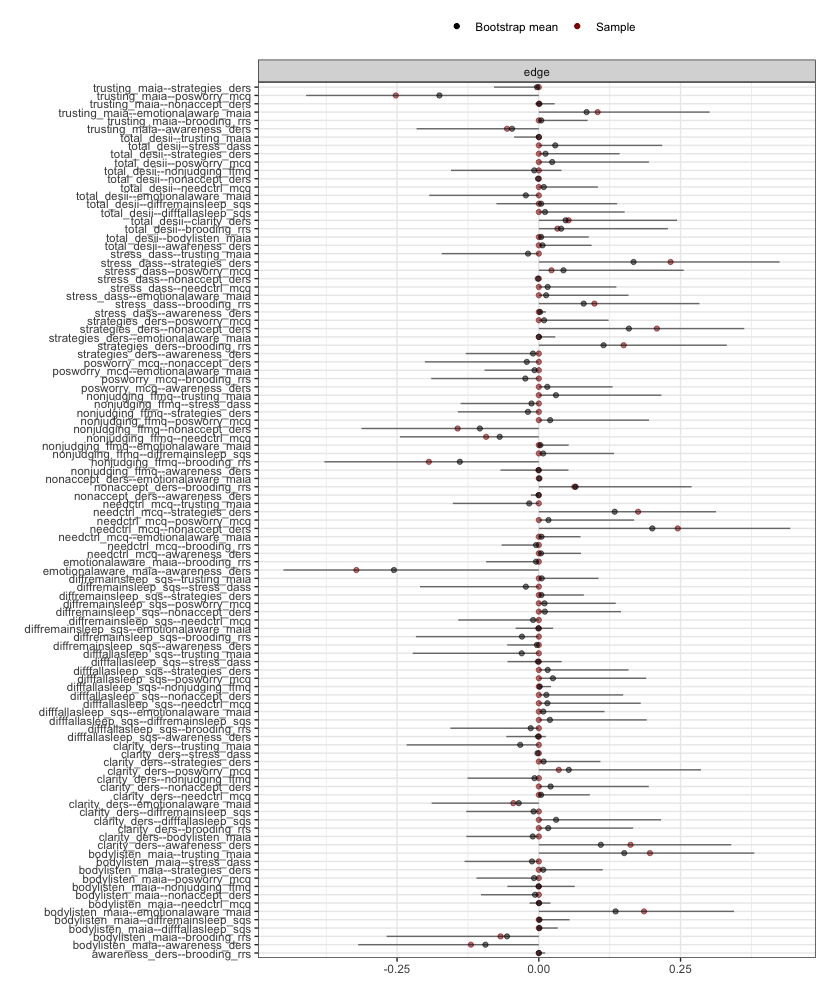


*Figure 4: Bootstrapped confidence intervals and means for symptom network weights*

Table 2: Edge weights, bootstrapped means, differences between edges and bootstrapped means, confidence intervals and cluster assignment for candidate symptoms evaluated for subsequent PLS-C analyses.

| Node1 | Node2 | Edge | Bootstrapped Mean | Difference | CI  (lower bound) | CI  (higher bound) | Cluster | Included? | Rationale for inclusion/exclusion |
| --- | --- | --- | --- | --- | --- | --- | --- | --- | --- |
| bodylisten_maia | brooding_rrs | -0.0675 | -0.0567854 | -0.0107146 | -0.2315793 | 0.09657933 | Bridge | YES | Bridge symptom, critical for representing cluster structure of network |
| nonjudging_ffmq | brooding_rrs | -0.1939165 | -0.1383415 | -0.055575 | -0.4299852 | 0.04215218 | Brooding | YES | Magnitude of bootstrapped mean and edge weight |
| stress_dass | brooding_rrs | 0.09810895 | 0.07668658 | 0.02142236 | -0.0785556 | 0.27477348 | Brooding | NO | Magnitude of bootstrapped mean and edge weight |
| strategies_ders | brooding_rrs | 0.14962884 | 0.1145427 | 0.03508613 | -0.0635981 | 0.3628558 | Brooding | YES | Magnitude of bootstrapped mean and edge weight |
| bodylisten_maia | awareness_ders | -0.1197583 | -0.0959271 | -0.0238312 | -0.3178382 | 0.07832166 | Mindfulness | NO | Magnitude of bootstrapped mean and edge weight  Large difference between edge weight and bootstrapped mean |
| bodylisten_maia | emotionalaware_maia | 0.18558621 | 0.13679369 | 0.04879252 | -0.0331636 | 0.40433603 | Mindfulness | NO | Magnitude of bootstrapped mean and edge weight |
| bodylisten_maia | trusting_maia | 0.1960093 | 0.14815254 | 0.04785676 | -0.0359993 | 0.42801788 | Mindfulness | YES | Magnitude of bootstrapped mean and edge weight |

##

## 1.4. Network centrality strength and betweenness

We additionally assessed network centrality (i.e., identifying which symptoms are most connected to others and are key to maintaining the network structure) and clustering (i.e., identifying groups of symptoms that are more strongly connected to each other than to other symptoms in other groups). We assessed centrality *strength* by summing edge weights for each node, and *betweenness* which quantifies how often one node is in the shortest paths between other nodes.

The symptom with the strongest centrality was “limited access to emotional regulation strategies” from the DERS, suggesting that this symptom is the most influential, or most influenced, irrespective of the mediating role of other symptoms. The symptom with the highest *betweenness* centrality index was unsurprisingly brooding, suggesting that this symptom is necessary to maintain the two clusters within this network.

*
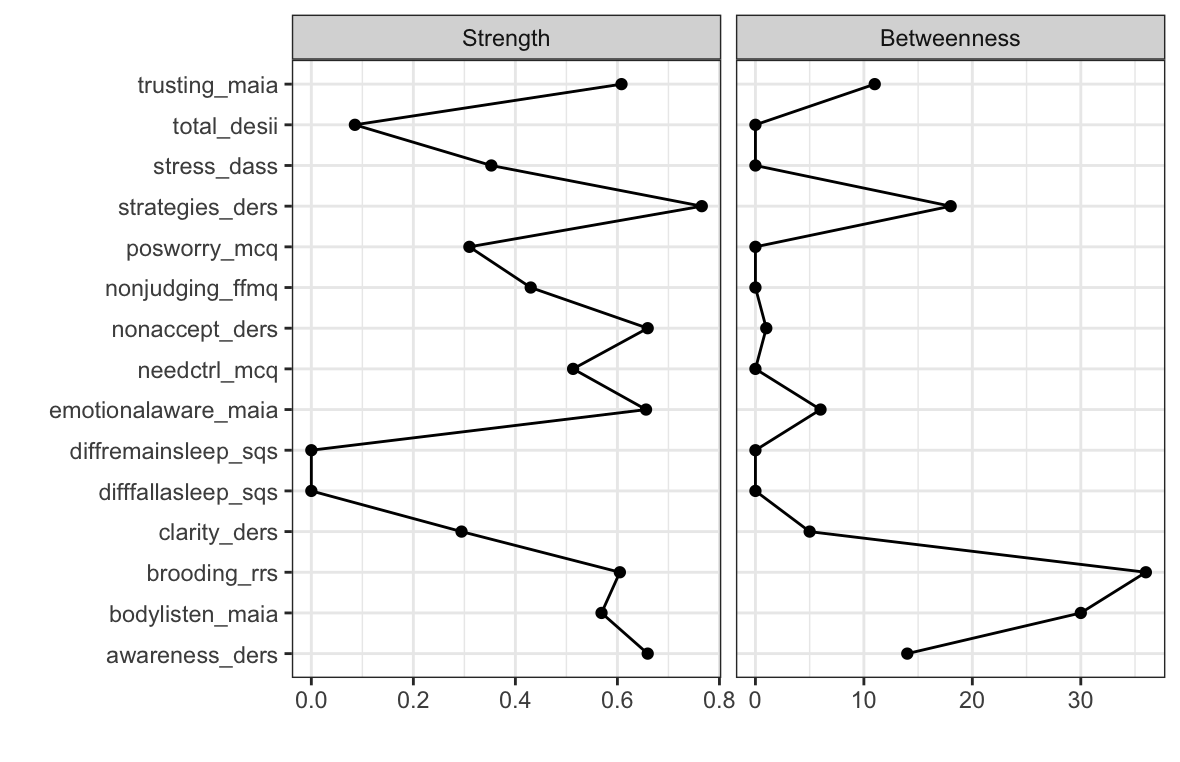
*

*Figure 5: Centrality indices for brooding symptom network and associated stability estimates.*

*
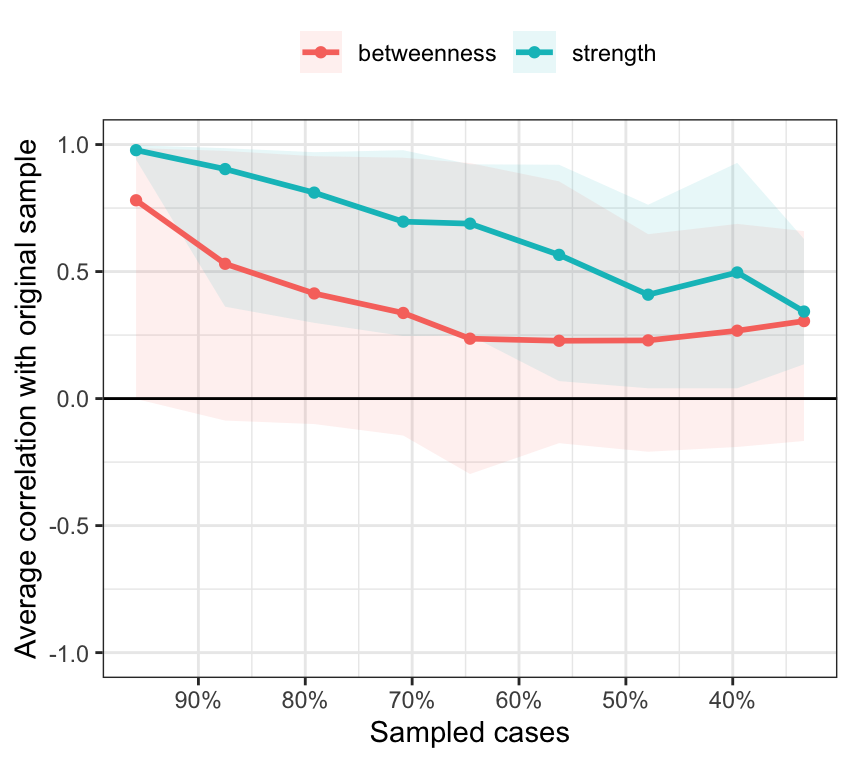
*

*Figure 6: Stability of centrality indices after case-dropping bootstrap (resampling without replacement). These results suggest that our indices are unstable, likely due to our small sample size.*
